# The Effects of Threonine Phosphorylation on the Stability and Dynamics of the Central Molecular Switch Region of 18.5-kDa Myelin Basic Protein

**DOI:** 10.1371/journal.pone.0068175

**Published:** 2013-07-05

**Authors:** Kenrick A. Vassall, Kyrylo Bessonov, Miguel De Avila, Eugenia Polverini, George Harauz

**Affiliations:** 1 Department of Molecular and Cellular Biology, University of Guelph, Guelph, Ontario, Canada; 2 Department of Physics, University of Parma, Parma, Italy; University of Oulu, Finland

## Abstract

The classic isoforms of myelin basic protein (MBP) are essential for the formation and maintenance of myelin in the central nervous system of higher vertebrates. The protein is involved in all facets of the development, compaction, and stabilization of the multilamellar myelin sheath, and also interacts with cytoskeletal and signaling proteins. The predominant 18.5-kDa isoform of MBP is an intrinsically-disordered protein that is a candidate auto-antigen in the human demyelinating disease multiple sclerosis. A highly-conserved central segment within classic MBP consists of a proline-rich region (murine 18.5-kDa sequence –T92-P93-R94-T95-P96-P97-P98-S99–) containing a putative SH3-ligand, adjacent to a region that forms an amphipathic α-helix (P82-I90) upon interaction with membranes, or under membrane-mimetic conditions. The T92 and T95 residues within the proline-rich region can be post-translationally modified through phosphorylation by mitogen-activated protein (MAP) kinases. Here, we have investigated the structure of the α-helical and proline-rich regions in dilute aqueous buffer, and have evaluated the effects of phosphorylation at T92 and T95 on the stability and dynamics of the α-helical region, by utilizing four 36-residue peptides (S72–S107) with differing phosphorylation status. Nuclear magnetic resonance spectroscopy reveals that both the α-helical as well as the proline-rich regions are disordered in aqueous buffer, whereas they are both structured in a lipid environment (*cf*., Ahmed *et al*., Biochemistry 51, 7475-9487, 2012). Thermodynamic analysis of trifluoroethanol-titration curves monitored by circular dichroism spectroscopy reveals that phosphorylation, especially at residue T92, impedes formation of the amphipathic α-helix. This conclusion is supported by molecular dynamics simulations, which further illustrate that phosphorylation reduces the folding reversibility of the α-helix upon temperature perturbation and affect the global structure of the peptides through altered electrostatic interactions. The results support the hypothesis that the central conserved segment of MBP constitutes a molecular switch in which the conformation and/or intermolecular interactions are mediated by phosphorylation/dephosphorylation at T92 and T95.

## Introduction

In the central nervous system (CNS), myelin arises from oligodendrocytes (OLGs), which proceed through a regulated pathway that assembles the components of the myelin membrane [1]–[5]. Myelination commences with differentiation of the bipolar early oligodendrocyte progenitor cell (OPC), and culminates with copious synthesis of classic myelin basic protein (MBP) isoforms and proteolipid protein (PLP), when extensive processes form and extend around an axon. Compact myelin is formed by flattening of the multiple, spirally-wrapped lamellae with the extrusion of cytoplasm, a process modeled in various ways, *e.g.*, the “liquid croissant” and “corkscrew” models [6]–[9]. The amount of white matter in the brain increases with evolutionary complexity [10]. Myelin continues to be formed until the early twenties in humans [11], and remodeling continues throughout adulthood in the healthy CNS [12]. Multiple sclerosis (MS) is a disease that is characterized by inflammatory demyelination of axons, for which the molecular mechanism has remained unknown over 150 years since its first major clinical documentation [13]. An “inside-out” model suggests that multiple sclerosis results from a cytodegenerative process aimed at the oligodendrocyte-myelin complex [14]–[16]: a process of gradual physical demyelination can then lead to an autoimmune response and a cycle of further degeneration, characteristic of the most common relapsing-remitting manifestation of multiple sclerosis. For many reasons, it is essential to attain an understanding of myelin formation and architecture at the molecular level in order to comprehend the causes and pathogenesis of this debilitating disease, as well as fundamental aspects of brain development and modeling.

**Figure 1:**
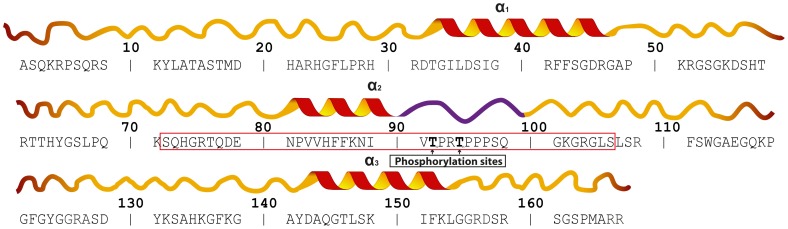
Amino acid sequence and secondary structural map of 18.5-kDa murine myelin basic protein. The protein is intrinsically-disordered in aqueous solution but has three distinct segments which have α-helical propensity as shown (α_1_, α_2_, α_3_) [27]. The sequence enclosed by the red rectangle represents the reference α_2_-peptide investigated in this study. This region consist of residues which can form an amphipathic α-helix when associated with lipids (α_2_), followed by a proline-rich region of the protein which adopts a left-handed poly-proline II structure (PPII, shown in purple) that is the probable SH3-ligand. Within this poly-proline structure are two mitogen-activated protein kinase sites at T92 and T95 [22]. The figure is modified from [49].

One of the most studied candidate auto-antigens in multiple sclerosis is the classic 18.5-kDa isoform of MBP, which is essential to the stability of central nervous system myelin where it plays numerous roles both in myelin development and homeostasis, acting both to adhere membrane leaflets to each other, and as a hub in protein-protein and protein-membrane interaction networks [17]–[22]. The latter include cytoskeletal proteins such as actin and tubulin, as well as signaling proteins such as calcium-activated calmodulin and SH3-domain containing proteins [23]–[25]. The murine 18.5-kDa MBP isoform has 168 amino acids, and is intrinsically disordered, like all members of this protein family. There are, however, three segments of the protein that become α-helical in the presence of lipids or membrane-mimetic solvents such as trifluoroethanol (TFE) [23], [26]–[28]. These three segments that undergo this specific disorder-to-order transition are denoted by us here as the α_1_-segment (murine 18.5-kDa residues T33-D46), α_2_-segment (P82-I90), and α_3_-segment (Y142-L154), respectively (Figure 1). In addition to being membrane-anchoring motifs, these segments can also “moonlight” as protein-protein interaction sites [22], [29].

*In vivo*, 18.5-kDa MBP undergoes extensive post-translational modifications [30], [31], which play a role in its ability to interact with a wide variety of partners [20]–[22]. One of the most important post-translational modifications in MBP is phosphorylation by mitogen-activated and other protein kinases (reviewed in [17], [22]). Phosphorylation of MBP is altered during development and ageing, and the overall level is decreased in multiple sclerosis [30], [32]–[34]. Phosphorylated MBP is associated with less compact myelin [35], and has been shown to be developmentally partitioned into detergent-resistant microdomains [36], [37]. The phosphorylation of MBP also protects it from proteases such as trypsin [38], which suggests that this modification alters the protein’s conformation [39]–[41]. *In vitro* MAP-kinase phosphorylation of 18.5-kDa MBP occurs sequentially at T92 and T95 (murine 18.5-kDa isoform numbering) [42]–[44], and modulates the ability of MBP to polymerise actin and tubulin, and to bundle microfilaments and microtubules [45]–[47]. Additionally, pseudo-phosphorylations (Thr to Glu substitutions) at these sites have been shown to affect the protein’s intracellular trafficking and its interactions with the non-receptor tyrosine kinase Fyn in transfected cells, and to inhibit the binding of MBP to the SH3-domain of Fyn *in vitro* [22], [24].

The T92 and T95 MAP-kinase sites in 18.5-kDa MBP are located within a central, proline-rich, highly-conserved region encoded by classic exons III and IV (Figure 1) [22]. This region can form a poly-proline type II (PPII) structure *in vitro*, and we have demonstrated that it represents an important protein interaction motif for SH3-domains such as those of Fyn [20], [22], [24], [25], [40], [48]–[50]. In all mammalian species, this proline-rich region in MBP is immediately adjacent to the α_2_-segment (Figure 1). The α_2_-segment represents a primary immunodominant epitope in multiple sclerosis; it associates with the phospholipid membrane as an amphipathic α-helix, presenting the PPII structure formed by the proline-rich region to the cytoplasm for potential protein-protein interaction [40], [48], [51], [52]. Previous molecular dynamics (MD) simulations by us demonstrated that phosphorylation at the MAP-kinase sites within the proline-rich region influences the interaction of this amphipathic α-helix with the membrane which could alter the orientation of the PPII structure in the cytoplasm [40].

Given that the MAP-kinase sites located within the proline-rich region of MBP are also only a few residues removed from the α_2_-segment, it is feasible that MAP-kinase phosphorylation could directly influence the conformation, dynamics, and stability of both the PPII and the α-helical structural elements. We have proposed that the central region of MBP encompassing the amphipathic α_2_-helical segment, and the adjacent PPII structure, constitute an important molecular switch in which membrane-MBP interactions, as well as MBP-protein interactions, are controlled by phosphorylation/dephosphorylation at the two MAP-kinase sites [20], [21], [40], [49], [50]. In this study, we seek to gain insights into the possible mechanisms of this molecular switch by probing the effects of phosphorylation at the MAP-kinase sites on the stability of the α_2_-helix, as well as on the conformation and dynamics of the PPII-structure. We have utilized four different 36-residue peptides (murine 18.5-kDa MBP residues S72-S107, denoted the α_2_-peptide) that contain the α_2_-helical segment (P82-I90) as well as the proline-rich segment (T92-S99), and that have varying phosphorylation status at T92 and T95 (Figure 1). We have determined and compared the free-energy change of disorder-to-α-helical transition in the unmodified and phosphorylated peptides using equilibrium TFE-titration curves monitored by circular dichroism (CD) spectroscopy. We also present new solution NMR spectroscopic data characterizing the structure of the unmodified α_2_-peptide in dilute aqueous conditions, building on our previous solution NMR spectroscopic studies in which the structure of the unmodified α_2_-peptide in phospholipid and lysophospholipid environments had been evaluated [49], [53], [54]. The spectroscopic experiments described here were complemented by MD studies on the identical unmodified and phosphorylated α_2_-peptides in water, as well as associated with dimyristoylphosphatidylcholine (DMPC) bilayers, that extend our previous MD experiments on shorter 24-residue peptides (murine 18.5-kDa residues E80-G103) [40], [49].

Overall, our results indicate that the MBP α_2_-peptide in aqueous environment does not readily form an α-helical structure, nor does it form PPII structure in the proline-rich region, in contrast to the α_2_-peptide in a lipid environment where both conformations are observed. Additionally, we find that phosphorylation at the MAP-kinase sites inhibits the formation of α-helical structure, and also affects the global conformation of the peptides through altered intramolecular electrostatic interactions. The results further our understanding of a critical region in 18.5-kDa MBP, and enhance our appreciation of possible consequences of aberrant amino acid substitutions or post-translational modification that may be involved in myelin destabilization during development [50], [55]–[57].

## Materials and Methods

### Expression, Purification, and Construction of α_2_-peptide Variants

Unlabeled as well as fully ^13^C-^15^N-labelled recombinant murine MBP 36-residue α_2_-peptide (S72–S107, murine 18.5-kDa sequence numbering – see Figure 1) was expressed as a SUMO-fusion, and purified by chromatography as described previously [23], [49]. This construct represented the (i) unmodified peptide variant. The synthetic phosphorylated variants of the α_2_-peptide were (ii) PhT92 (phosphorylation at residue T92), (iii) PhT95 (phosphorylation at T95), and (iv) PhT92–PhT95 (double phosphorylation at residues T92 and T95), and were purchased from Biomatik (Cambridge, ON). All other materials were as previously described [49].

### Solution NMR Spectroscopy of the Recombinant α_2_-peptide in Aqueous Buffer

Unmodified α_2_-peptide samples for solution NMR spectroscopy were prepared by dissolving ∼2 mg of uniformly ^13^C-^15^N-labelled α_2_-peptide in a solution containing 100 mM NaCl, 20 mM HEPES-NaOH (pH 7.5), and 10% D_2_O. The final sample volume was ∼600 μL, and the sample was maintained at 295K for all measurements.

### Sequence-specific Resonance Assignments and Structure Calculations

High-resolution ^1^H, ^13^C, and ^15^N NMR spectra, using several complementary pulse sequences [58], were recorded on a Bruker Avance spectrometer operating at a Larmor proton frequency of 600.13 MHz. In order to obtain residue-specific assignments, a two-dimensional ^1^H-^15^N-HSQC (heteronuclear single-quantum coherence) experiment was conducted in addition to several triple-resonance NMR experiments as follows: (a) HNCO/HN(CA)CO to assign the amide proton (^1^H_N_[*i*]), the amide nitrogen (^15^N[*i*]), and the carboxyl carbon atoms of the current and preceding amino acid (^13^Ć[*i*], ^13^Ć[*i-1*]); (b) CBCA(CO)NH/HNCACB to assign the C_α_ and C_β_ atoms of the current and preceding amino acids; (c) HCCH-TOCSY (total correlation spectroscopy) to assign the remaining ^1^H and ^13^C side-chain atoms; (d) HACAN to assign the prolyl residues [59]; and (e) two-dimensional ^1^H-^13^C-HSQC spectra without carbon decoupling to extract heteronuclear ^1^J_CαHα_ coupling constants, which allows for differentiation of the PPII and random coil conformations [60]. Water suppression was achieved using the double-pulsed field gradient spin echo technique (excitation sculpting) with the carrier frequency set to the water ^1^H signal. Detailed experimental spectroscopic parameters are given in Table 1 [61]–[63].

**Table 1:** Summary of solution NMR acquisition parameters.

Spectra	Acquisition Parameters											
	Transients	Carrier Frequency (ppm)			Sweep Width (ppm)			Number of Points			Quadrature Detection^a^	
		*F_1_*	*F_2_*	*F_3_*	*F_1_*	*F_2_*	*F_3_*	*F_1_*	*F_2_*	*F_3_*	*F_1_*	*F_2_*
^13^C-HSQC	8	40	4.7	–	100	10	–	256	2048	–	E-A-E	–
HCAN	8	120	54	4.7	44	32	16	72	88	2048	S-TPPI	**S-TPPI**
HCCH	8	40	40	4.7	80	80	14	88	80	2048	S-TPPI	**S-TPPI**
HNCACB	8	40	118	4.7	64	25	14	128	40	2048	S-TPPI	**S-TPPI**
CBCA(CO)NH	8	40	118	4.7	64	25	14	128	40	2048	S-TPPI	**S-TPPI**
HNCO	24	175	118	4.7	16	25	14	64	40	2048	S-TPPI	**S-TPPI**
HN(CA)CO	24	175	118	4.7	16	25	14	64	40	2048	S-TPPI	**S-TPPI**
**^15^N-HSQC**	**8**	**120**	**4.7**	**–**	**80**	**14**	**–**	**256**	**2048**	**–**	**E-A-E**	**–**

(S-TPPI = States-TPPI, E-A-E = Echo-Anti-Echo) [61]–[63].

**Figure 2:**
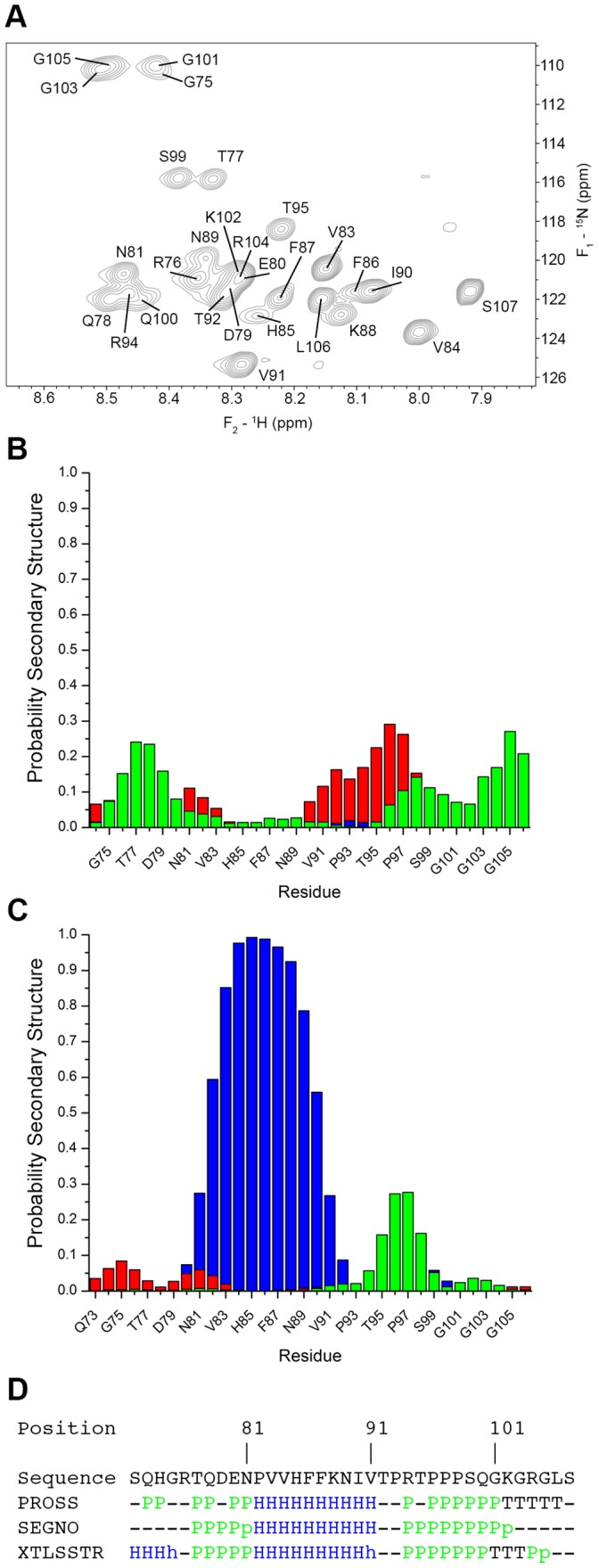
Nitrogen-HSQC NMR spectra and secondary structure analysis of the MBP α_2_-peptide. (A) The ^1^H-^15^N HSQC spectrum of uniformly ^13^C-^15^N-labelled α_2_-peptide (S72-S107) dissolved in 20 mM HEPES-NaOH, 100 mM NaCl, and 10% D_2_O at a concentration of 1.47 mM, and recorded at 295 K. A total of 29 of 31 expected backbone peaks were assigned (there are 5 prolyl residues). The HSQC spectrum was processed by applying a 90^o^-shifted squared-sine bell function, and zero-filled up to 256 and 2048 complex points along *F*_1_ and *F*_2_, respectively, prior to Fourier transformation using NMRPipe. (B) Prediction of secondary structure probabilities for each residue (populations per residue) in the α_2_-peptide in aqueous solution, using a method designed for disordered proteins [68]. The method differentiates between α-helix (blue), β-sheet (red), PPII (green), and random coil (not shown). The probabilities were calculated using the H_α_, H_N_, C_α_, C_β_, C′, and N chemical shift assignments for the α_2_-peptide. (C) Prediction of secondary structure probabilities for each residue in the α_2_-peptide in the presence of DPC, using a method designed for disordered proteins [49], [68]. The H_α_, H_N_, C_α_, C_β_, C′, and N chemical shift assignments deposited in BMRB 6100 were used to calculate the secondary structure probabilities. (D) Secondary structure assignment methods used on the α_2_-peptide in the presence of DPC (PDB ID 2LUG). The XTLSSTR [91], PROSS [92], and SEGNO [93] methods all take into consideration PPII conformations, and differentiate them from coil, α, and β structures. The PROSS and SEGNO results were calculated using the “*Polyproline*” server created by the DSIMB bioinformatics team. The XTLSSTR results were obtained via the 2struc server created by the Wallace Laboratory [109]. The assignment indicates that the proline-rich region has a PPII conformation.

The ^1^H chemical shifts were referenced directly to the methyl signal of DSS (2,2-dimethylsilapentane-5-sulphonic acid) in an external sample tube, whereas the ^13^C and ^15^N chemical shifts were referenced to DSS indirectly. The spectra were processed using NMRPipe [64]. All free induction decays were zero-filled, and apodized using a shifted, squared sinusoidal bell function prior to Fourier transformation and subsequent phase-correction. The ^1^H-^15^N-HSQC spectrum was zero-filled up to 2048 and 4096 complex points along *F*_1_ and *F*_2_, respectively (Figure 2A). The HN(CA)CO, HNCACB, and their complementary spectra were zero-filled up to 256, 256, and 2048 complex points along *F*_1_, *F*_2_, and *F*_3_, respectively. The HACAN spectrum was zero-filled up to 256, 256, and 2048 complex points along *F*_1_, *F*_2_, and *F*_3_, respectively. The ^1^H-^13^C-HSQC spectrum was zero-filled up to 4096 complex points along each of *F*_1_ and *F*_2_. The acquisition parameters for all spectra are listed in Table 1.

Spin systems were then assigned using Computer Assisted Resonance Assignment (CARA, version 1.8.4) [65], modules contained in the CARA software package (www.nmr.ch), and a collection of in-house scripts that have been previously described [49], [66]. All spin systems were created on the basis of the ^13^C, ^15^N, and ^1^H ppm values of ^13^Ć[*i*-1], ^15^N[*i*], and ^1^H_N_[*i*] of each system in the HNCO spectrum. All other spin systems were picked and partially assigned automatically using another set of in-house scripts. Spin systems that were not identified by the scripts were assigned manually. Sequence-specific connectivity was obtained manually using iterative trials.

Secondary structure estimation by chemical shift index (CSI) analysis was performed as described [67]. The assignments (H_α_, C_α_, C_β_, C′, N, H_N_) were also input into the Vendruscolo Laboratory’s δ2D software, which uses an algorithm designed to calculate the probability that any amino acid in a primarily disordered protein has a particular secondary structure conformation, based on the chemical shift assignments [68]. The chemical shift data have been deposited into the Biological Magnetic Resonance Bank (BMRB ID 19186).

### Forward and Reverse TFE-titration Curves of the α_2_-peptide

The four peptides used in this study were each titrated with trifluoroethanol (TFE) by diluting concentrated peptide stock (typically ∼130 µM peptide) in water into different solutions containing successively higher TFE concentrations. The final solution conditions in all cases were: 15 µM peptide, 20 mM HEPES-NaOH, pH 7.4, and TFE concentrations ranging from 0 M to ∼7.7 M (0 to 55%, v/v). All samples were incubated for ∼16 hours at 25°C prior to measurement.

The TFE-titration experiments were monitored by CD using a Jasco J-815 spectropolarimeter (Japan Spectroscopic, Tokyo, Japan) at a fixed wavelength of 222 nm using a quartz cuvette with a 1-mm path length, thermostatted at 25°C using a Jasco PTC-424S/15 Peltier temperature controller (Japan Spectroscopic, Tokyo, Japan). At this wavelength and temperature, based on buffer measurements, the non-peptide components of the solution gave virtually no CD signal, and the measured ellipticity was due exclusively to the peptide. A total of 30 readings of each sample were taken over a 30-second period (*i.e.*, one per second), and averaged in order to reduce scatter in the curves. At least 3 independent TFE-titration curves of each peptide were measured, with each curve analyzed individually (see below).

Reverse TFE-titration curves of the peptides were also measured by making up a concentrated peptide solution in high TFE (∼50% v/v), followed by dilution to lower TFE concentrations. The reverse-titration samples were incubated and measured in the same way as the forward-titration curves.

### Quantitative Analysis of TFE-titration Curves

The TFE-titration curves of the four α_2_-peptide variants were fit to a 2-state equilibrium model: disordered↔α-helical, with equilibrium constant *K^TFE^*. For this transition, a linear dependence of Δ*G* on TFE concentration was assumed [69], [70], allowing the equilibrium constant to be defined by:
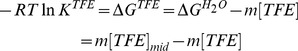
(1)where *R* is the universal gas constant, *T* is the temperature in Kelvin, Δ*G^TFE^* is the free energy of the transition at a given concentration of TFE, 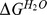 is the free energy of the transition in the absence of TFE (*i.e.*, in dilute buffer), *m* is a measure of the dependence of Δ*G^TFE^* on TFE concentration, and [*TFE*]*_mid_* is the concentration of TFE at which the disorder-to-helical transition is half-completed. The data were fit to the following equation as previously described for a 2-state equilibrium process [71], [72]:
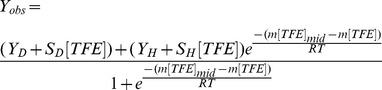
(2)where, Yobs is the observed CD signal, YD and YH are the y-intercepts of the pre-transition and post-transition baselines, respectively, and SD and SH are the slopes of the pre-transition baseline (between 0 M and ∼1 M TFE), and the post-transition baseline (above ∼4 M TFE), respectively.

Fitting to Equation (2) was done using Microcal Origin version 8 (Northampton, MA). In order to reduce the number of fitted parameters, and to determine the values of *m* and [*TFE*]*_mid_* more precisely, *Y*_D_, *S_D_*, *Y_H_*, and *S_H_* were determined by linear regression analysis, and were then set as fixed parameters in non-linear curve fitting. All values are reported as an average of at least 3 independent experiments, and reported errors are standard deviations.

### Molecular Dynamics (MD) Simulations of the MBP α_2_-peptides

The GROMACS 4.5.5 software package [73] with the Gromos96 ffG53a6 force-field [74] was used to perform molecular dynamics (MD) simulations using the SHARCNET high performance computer cluster (www.sharcnet.ca). Peptide models used in simulations were constructed from the lowest energy structures obtained from our solution NMR experiments in dodecylphosphocholine (DPC) micelles [49] (PDB ID 2LUG). This structure was used essentially without modification except for the addition of PO_4_^2-^ groups to the T92 and T95 residues as appropriate using the SYBYL-X 1.3 molecular modeling suite (SYBYL, Tripos Associates, St. Louis, MO). The following 4 models were considered: (i) unmodified, representing the original solution NMR structure; (ii) singly-phosphorylated at Thr92 only (PhT92); (iii) singly-phosphorylated at Thr95 only (PhT95); and (iv) doubly-phosphorylated at both Thr92 and Thr95 (PhT92-PhT95).

All four peptides were simulated in H_2_O, as well as in a DMPC lipid bilayer system, as described below. Both the N- and C-termini of the peptide were uncharged, and all histidyl side chains were unprotonated. All peptide bonds were in the *trans* conformation. All simulations in DMPC were done in duplicate, whereas the simulations in water were done in triplicate due to their increased dynamics and variability under these conditions.

#### Molecular dynamics simulations in H_2_O

The four α_2_-peptides with varied phosphorylation states were simulated at 37°C in a cubic virtual box with dimensions 14×14×14 nm. Each peptide was positioned in the center of the box, and the box was subsequently solvated with water molecules using the spc216 model [75]. The final density of the system was 997.2 g/L. To obtain an overall net charge of zero, Na^+^ or Cl^-^ counter-ions were added as appropriate. Subsequently, the solvated and neutralized system was energy-minimized to a maximum overall force of <1,000 kJ/mol/nm using the steepest descent minimization algorithm with the *rcoulomb* and *rvdv* cut-offs set at 1.2 nm. The equilibration steps were done at 1 atm and 310 K, using Berendsen isotropic pressure coupling and velocity rescaling with a stochastic term (*v-rescale*) thermostat for temperature coupling [76]. For the production MD runs, the time step (*dt*) was set to 0.002 ps, and the same pre-equilibration settings were used with respect to temperature and pressure couplings. The equilibrated system was simulated for total of 160 ns.

#### Molecular dynamics simulations in DMPC bilayers

Each of the four α_2_-peptide variants was also simulated at 37°C in the presence of a DMPC lipid bilayer, surrounded by H_2_O with counter-ions on both sides; a system that we have used previously [40]. The peptides were initially positioned horizontally on top of the bilayer according to previous experimental data, with the hydrophobic residues F86 and F87 pointing towards the membrane [20], [40], [51]. In order to position the peptide relative to the DMPC bilayer correctly, the peptide was first put in a vacuum box of the same dimensions as the DMPC lipid system. The box containing the peptide was merged with the DMPC reference membrane, and potential steric clashes were eliminated automatically using *genbox* with the default van der Waals radii distance threshold of 0.105 nm. The final merged simulation box had a volume of 828 nm^3^ with a density of 955 g/L; an overall net charge of zero for the system was obtained by adding Na^+^ or Cl^-^ counter-ions, as appropriate.

The peptide-DMPC simulation box was then energy-minimized using the steepest descent algorithm to a final maximal force of 1,000 kJ mol^−1^ nm^−1^, and this procedure was followed by two equilibration steps in which the peptide was restrained to avoid potential structural distortion. The first equilibration step was carried out for 100 ps at constant number of particles, volume, and temperature (NVT). The second equilibration step was carried out for 1,000 ps at constant number of particles, pressure, and temperature (NPT). These equilibration steps were important for allowing the lipids and water molecules to accommodate the structured peptide without significant unfolding.

The equilibrated and energy-minimized system was simulated using a 0.002 ps time step for a total of 160 ns at 37°C and 1 atm. All equilibration and simulation steps were thermally- and pressure-coupled using the same parameters as for the H_2_O simulations described in the previous section.

#### Molecular dynamics simulations with varying temperature

The thermal unfolding propensity of the four energy-minimized and equilibrated structures of the unmodified, PhT92-, PhT95-, and PhT92-PhT95-α_2_-peptides associated with the DMPC lipid bilayer were measured qualitatively using temperature ramps (*i.e.*, simulated annealing). Due to disruption of the DMPC bilayer at high temperatures, only the peptide was subjected to the temperature-ramp protocol, while keeping the DMPC bilayer, counter-ions and H_2_O molecules at a constant temperature of 37°C throughout these experiments. These temperature-ramp simulations were performed for a total of 25 ns and involved, sequentially: (i) a linear gradient from 37°C to 500°C during the first 10 ns (temperature ramp of 46.3°C/ns), followed by (ii) maintenance at 500°C for a total of 5 ns, and finally (iii) a linear decrease at a rate of 46.3°C/ns in temperature from 500°C back to 37°C, over a 10-ns time interval. The same temperature and pressure coupling settings that were used in the isothermal H_2_O and DMPC simulations were also applied in these temperature-ramp experiments, with a time step of 0.002 ps. The suitability of this protocol for assessing differences in α-helix stability of the peptides was assessed by performing validation experiments in which all the residues within the α-helical segment (P82-I90) of the unmodified peptide were mutated to alanine, valine, and glycine respectively, followed by temperature-ramping using the exact same protocol as outlined above. The results of these validation experiments revealed that the poly-alanine helix denatured at a higher temperature, followed by the poly-valine and poly-glycine helices (see Figure S4), as expected based on differences in α-helical propensity of these residues [77].

### Analysis of MD Experiments

The 160-ns trajectories from the H_2_O and DMPC bilayer simulations were analyzed frame by frame (each frame equivalent to 10 ps) using GROMACS utilities. Secondary structure evolution was evaluated with the dictionary of protein secondary structure (DSSP) algorithm [78]. The tilt dynamics of the α-helix (residues P82-I90) within the α_2_-peptide (residues S72-S107– see Figure 1) with respect to the DMPC bilayer surface were evaluated. The tilt is defined as the angle Θ between the helix axis passing through its center of mass, and the axis parallel to the plane of the phospholipid head groups of the membrane.

The dihedral angles of each residue in the peptide, as well as the membrane penetration depth of the α-helix, were determined using VMD software [79] and custom scripts written in the TCL language. In the calculation of membrane-penetration depth, the surface plane of the leaflet was defined by selecting the phosphates of the DMPC lipids, and the geometrical center of the α-helical segment of the peptide was determined using the α-carbons of the amino acid residues. Using this information, the membrane-penetration depth was thus expressed as the distance between the center of mass of the α-helix and the surface of the membrane. The presence of PPII structure in the peptides over the 160-ns trajectory was determined by creating a classifier function based on defined φ and ψ angle thresholds. Peptides were classified to adopt a PPII structure if at least 2 adjacent residues satisfied the following criteria as previously defined [80]: (a) −46° ≤φ ≥ −104°, (b) 116° ≤ ψ ≥174°, and (c) ω = 0.

## Results

### Solution NMR Spectroscopy of Recombinant α_2_-peptide

Previously, we have evaluated the conformation of the 36-residue α_2_-peptide (murine 18.5-kDa residues S72–S107) in the presence of DPC micelles, and found that the peptide adopted a well-defined amphipathic α-helix in the region P82-I90, whereas the remainder of the protein was largely disordered [49]. Separate CD experiments conducted on this peptide in aqueous conditions [23] (see also Figure S3), indicated that although the central α-helical structure is much less well defined, there could be some residual secondary structure present under these conditions. This result was consistent with solution NMR data obtained on the entire 18.5-kDa protein [27]. Here, we have performed several solution NMR experiments on the α_2_-peptide under aqueous conditions in order to characterize better any residual secondary structure, as well as to define more precisely the conformation of the proline-rich region, which is the probable SH3-ligand [24], [48], [49].

Consistent with the overall intrinsically-disordered nature of the protein, the nitrogen-HSQC spectra obtained under aqueous conditions showed a high degree of degeneracy (Figure 2A), and so additional experiments were needed in order to make full resonance assignments. We collected a series of three-dimensional and two-dimensional spectra, and used the same assignment strategy as previously for the α_2_-peptide in DPC micelles [49]. These data allowed us to resolve the degeneracy observed in the HSQC spectrum, and to obtain nearly complete ^1^H, ^15^N, and ^13^C backbone assignments for the peptide. The ^1^H_α_, ^13^C_α_, and ^13^C_β_ resonances were completely assigned, with the exception of residues S72 and Q73, which were not identified in any spectra. These assignments were used to confirm sequential connectivity of the residues. A complete list of resonance assignments is given in Table S1.

Several different algorithms were used to identify any propensities of the α_2_-peptide to form secondary structural elements. Based on the C_α_ chemical shift deviations from random coil values, we found that the peptide does not have any obvious secondary structural elements, given that there are no two sequential residues that are ±0.7 ppm of the established random coil values (Figure S1) [67], [81], [82]. The deviations were all sequence-corrected [83].

The secondary structure propensity (SSP) algorithm designed by the Forman-Kay group and collaborators was also used to determine secondary structure elements in the unmodified α_2_-peptide [84]. An SSP score of 1 indicates that the residue is in an α-helical conformation, whereas −1 suggests β-structure. To account for the intrinsically-disordered nature of the α_2_-peptide, only the ^1^H_α_, ^13^C_α_, and ^13^C_β_ chemical shifts were considered for the calculations based on the recommendations of the designers. This algorithm also determined that the α_2_-peptide had a high propensity to remain in the random coil conformation under these conditions, as most scores remained close to zero (Figure S2). Dihedral angle determination by TALOS+ [85] also found the torsion angles of the α_2_-peptide to be dynamic, with high deviations from the predicted ψ and φ angles for most of the residues (Table S2).

One last algorithm was also used to determine secondary structure propensities. The δ2D algorithm designed by the Vendruscolo laboratory outputs the probability of each residue adopting α-helical, β-strand, PPII, or random coil conformation [68] (Figure 2B). The results suggested a high percentage of random coil throughout the peptide, with up to ∼25% probability of PPII conformation at its N- and C-termini. The probability of PPII conformation within the proline-rich region was surprisingly near nil, and there was an added likelihood of β-structure (up to 30% probability) present in this region. It should be noted that the percentages are different from those obtained in DPC [49] (Figure 2C), where the highest probability of PPII conformation (up to ∼25%) is located directly in the proline-rich region, with very little propensity for PPII conformation observed elsewhere.

As a complement to these secondary structure analyses based on chemical shifts, we also used coupling constants, namely ^1^J_CαHα_ to differentiate between random coil and PPII conformations [60]. In this method, the PPII conformation can be differentiated by observing the amide nitrogen deviation from random coil values, as well as the deviation from random coil values of coupling constants. We calculated the ^1^J_CαHα_ coupling constants for the region that spanned residues V91-S99 (Table 2). This segment encompasses the proline-rich region of the α_2_-peptide, and was selected because it was the only segment that fulfilled the criteria of having sequential residues with a deviation in the ^15^N resonance >1.1 ppm of the corresponding random coil value [60]. Given that the calculated coupling constants were almost all within 1.1 Hz of the random coil values for the respective residues, the torsion angles associated with the amino acids in this region suggest a random coil conformation.

**Table 2:** Comparison of experimental coupling constants to established random coil values for the residues in the proline-rich region [63].

Residue	^1^J_HαCα_ (Hz)	Random Coil ^1^J_HαCα_ (Hz)	Δ^1^J_HαCα_ (Hz)	Δδ^15^N (ppm)
V91	141.62	141.3	0.32	1.13
T92	141.02	141.4	−0.38	3.394
P93	148.83	148.4	0.43	4.929^a^
R94	141.62	141.5	0.12	2.039
T95	141.02	141.4	−0.38	2.986
P96	144.02	148.4	−4.38	6.288^a^
P97	146.43	148.4	−1.97	2.349^a^
P98	147.63	148.4	−0.77	2.588^a^
S99	142.82	142.1	0.72	−0.79

a The amide nitrogen random coil chemical shift value was extracted from Koehler *et al*. [110].

### Analysis of CD Spectroscopic TFE-titration Curves

The propensity of the largely disordered α_2_-peptide to adopt α-helical structure was evaluated by performing quantitative TFE-titration curve experiments, as has been done for other proteins and peptides [69], [70], [86]. Fitting of the titration curve data to obtain the change in free energy (Δ*G*) of the disorder↔α-helical transition defines the thermodynamic feasibility of α-helical formation, with low values of Δ*G* suggesting a more stable α-helical conformation. The MAP-kinase phosphorylation sites in MBP are at residues T92 and T95, immediately adjacent to the C-terminal end of the α-helical segment, but it is unclear whether phosphorylation at these sites has an effect on the stability of the α-helix. We had not specifically addressed the stability question *per se* in our previous MD study [40].

**Figure 3:**
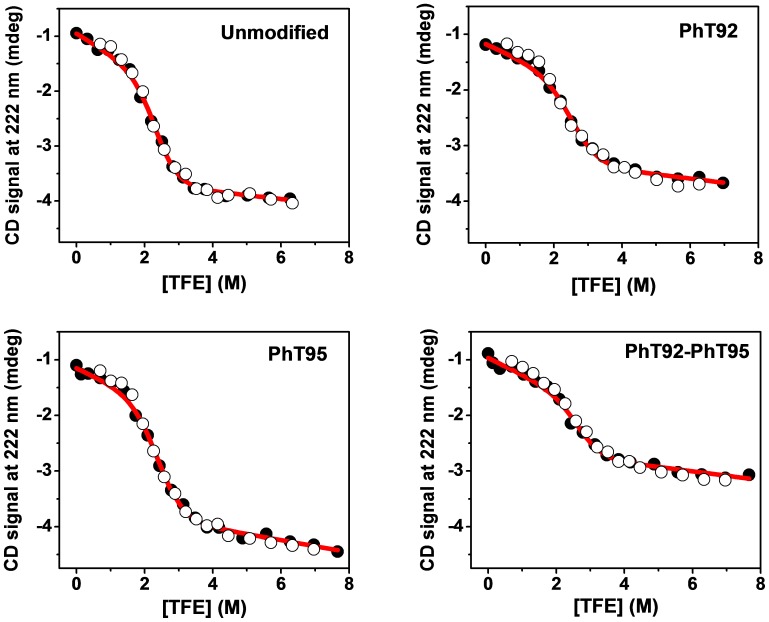
CD-monitored trifluoroethanol-titration curves of α_2_-peptide variants of 18.5-kDa MBP. Data are presented for four α_2_-peptides (S72–S107) of myelin basic protein (MBP): unmodified, singly- (PhT92, PhT95) and doubly- (PhT92–PhT95) phosphorylated. The peptides were titrated with TFE, and monitored by CD spectroscopy at 222 nm in order to assess the differences in their ability to form α-helical structure. All samples contained 20 mM HEPES-NaOH, pH 7.4, and data were collected at 25°C. The solid circular points are representative data of a forward-titration experiment, whereas the open circular points are from the corresponding reverse-titration experiments. The reverse-titration data were offset to match pre- and post-transition baselines to facilitate comparison. Forward- and reverse-titration data are highly coincident which is indicative of an equilibrium folding transition amenable to thermodynamic analysis. The red lines are fits of the forward-titration data to a 2-state (disorder↔α-helix) equilibrium model [69] to obtain the midpoint ([*TFE*]*_mid_*) of the curves, as well as the Δ*G^H2O^* of the transition (Table 3).

The CD-monitored TFE-titration curves of all four α_2_-peptides (unmodified, PhT92, PhT95, and PhT92–PhT95) are sigmoidal, with a single pronounced transition, consistent with a cooperative 2-state folding process that would be expected for a peptide of this size (Figure 3). A 2-state process is also suggested by the presence of an isodichroic point in full CD spectra (Figure S3) at low wavelengths (∼202.5 nm for unmodified and ∼1–2 nm lower for the variants) [87]–[89]. Reverse-titration curves, in which the α_2_-peptide in the presence of high TFE concentration is diluted to lower TFE concentration, are coincident with the forward-titration curves, indicating a reversible transition that can be analyzed in thermodynamic terms (Figure 3).

The TFE-titration data were fit well to a 2-state equilibrium model [69], [70] (disordered↔α-helical) defining Δ*G^H2O^* (free energy of transition in the absence of TFE, *i.e.*, in aqueous buffer) and [*TFE*]*_mid_* (the concentration of TFE at which the transition is 50% completed) (Figure 3, Table 3). The [*TFE*]*_mid_* of the unmodified peptide was found to be 2.31 M (∼17% v/v), whereas the Δ*G^H2O^* is 14.9 kJ mol^−1^. The positive value of Δ*G^H2O^* suggests that α-helical formation in aqueous conditions (in the absence of TFE) is unfavorable, which is consistent with the observation of very little α-helical content under these conditions by CD (Figure S3) and NMR spectroscopy (Figure 2). The changes in the [*TFE*]*_mid_* and Δ*G^H2O^* values resulting from phosphorylation can be represented as Δ[*TFE*]*_mid_* (determined as [*TFE*]*_mid_* of phosphorylated variant – [*TFE*]*_mid_* of unmodified peptide), and ΔΔ*G^H2O^* (calculated as Δ*G^H2O^* of phosphorylated variant – Δ*G^H2O^* of unmodified peptide) (Table 3). The three phosphorylated variants all have positive Δ[*TFE*]*_mid_* and ΔΔ*G^H2O^* values, ranging from ∼0.2–0.4 M and ∼1.4–2.5 kJ mol^−1^, respectively (Table 3). The data thus suggest that phosphorylation at the MAP-kinase sites in MBP generally tend to disfavor formation of the α-helical-rich conformation, especially when phosphorylation occurs at residue T92.

**Table 3:** Thermodynamic parameters for the disorder ↔ α-helix transition within the MBP α_2_-peptides in aqueous conditions.

α_2_-peptide variant	a[*TFE*]*_mid_* (M)	bΔ[*TFE*]*_mid_* (M)	cΔ*G^H2O^* (kJ/mole)	dΔΔ*G^H2O^* (kJ/mole)
Unmodified	2.31±0.07	n.a	14.9±0.5	en.a
PhT92	2.65±0.09	0.3	17.2±0.6	+2.2
PhT95	2.53±0.10	0.2	16.4±0.7	+1.4
PhT92–PhT95	2.69±0.03	0.4	17.4±0.2	+2.5

Thermodynamic values were determined at 25°C from fitting CD-monitored TFE-titration curves to a 2-state transition (disordered↔α-helical). All errors (±) are standard deviations from at least three independent experiments.

a [*TFE*]*_mid_* was determined from data fitting and represents the [TFE] at the mid-point of the transition (*i.e.*, [TFE] when half the peptide molecules contain an α-helical conformation).

b Δ[*TFE*]*_mid_* = [*TFE*]*_mid_* (phosphorylated peptide)−[*TFE*]*_mid_* (unmodified peptide).

c Δ*G^H2O^* is the change in free energy of the transition calculated as Δ*G^H2O^* = [*TFE*]*_mid_*•*m*; where *m* is the dependence of Δ*G* on [TFE], determined directly by independently fitting all titration curves (both modified and phosphorylated) and averaging to yield a value of 6.5±0.7 kJ mole^−1^ M^−1^.

d ΔΔ*G^H2O^* = Δ*G^H2O^* (phosphorylated peptide) − Δ*G^H2O^* (unmodified peptide).

e n.a, not applicable.

### Molecular Dynamics Simulation Experiments

**Figure 4:**
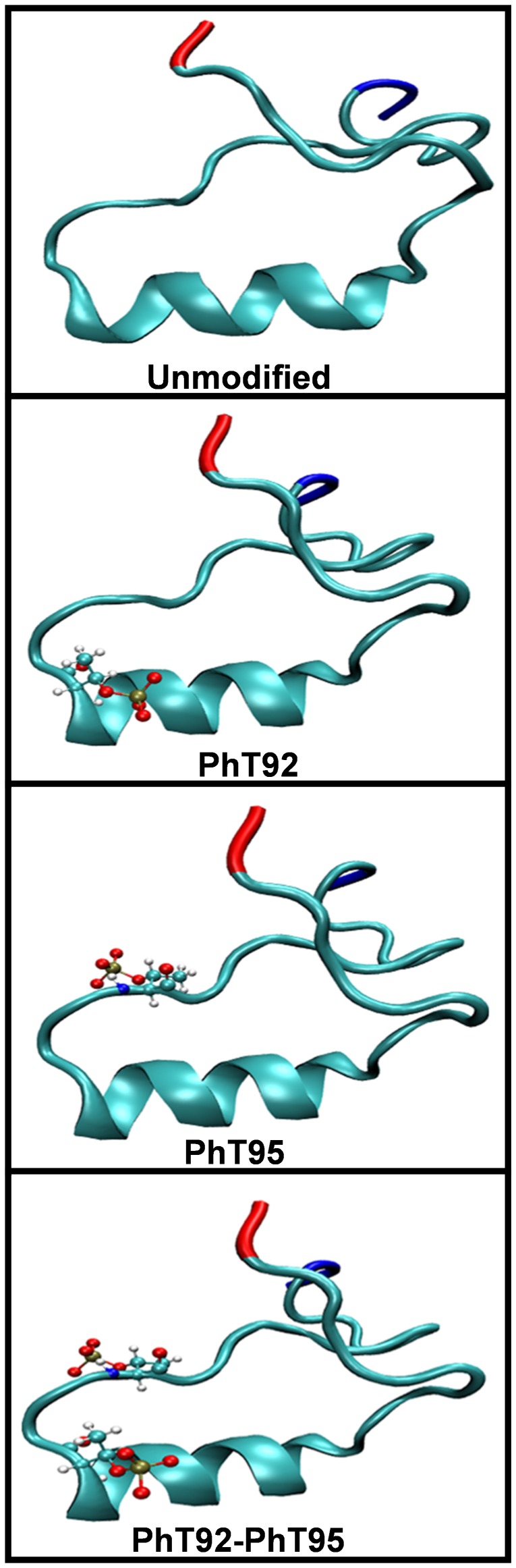
The starting structures of the MBP α_2_-peptides used in molecular dynamics simulations. The structures were derived from NMR data [49] and energy-minimized before MD experiments. The four α_2_-peptides (S72–S107) of myelin basic protein (MBP) used in this molecular dynamics study are: the unmodified α_2_-peptide, as well as the singly-phosphorylated α_2_-peptides at residues T92 and T95 (PhT92 and PhT95 respectively), and the corresponding doubly-phosphorylated α_2_-peptide (PhT92–PhT95). The N- and C-termini are designated by red and blue colors, respectively.

The one unmodified and three phosphorylated 36-residue α_2_-peptide variants were simulated in water as well as in DMPC bilayers at 37°C for 160 ns. These experiments expand upon our previous MD experiments on shorter 24-residue peptides (murine 18.5-kDa residues E80–G103) [40], [49]. In these 24-residue peptides, the residues E80–V91 were modeled as an α-helix, whereas the remaining residues T92-G103 were initially modeled as an extended PPII structure to provide a starting point, since experimental structural coordinates were not available at that time. In the MD experiments presented here, the starting structures were derived from NMR experiments that determined the structure of the α_2_-peptide in the presence of DPC micelles [49], with little modification (Figure 4). Given that the DPC and DMPC lipidic systems have similar characteristics, this NMR structure elucidated in DPC is expected to be a good starting structure for MD simulations. The starting structures of the α_2_-peptides used here have a slightly shorter α-helical segment (P82-I90) than previously modeled in the starting structures of the 24-residue peptides. Additionally, the PPII structure formed by residues in the proline-rich region in the α_2_-peptide is shorter compared to the modeled 24-residue peptide (comprising ∼7 residues versus 12 residues) (Figure 2C and 2D). Moreover, the N-terminal region (S72–V83) and the C-terminal region (T92-S107) of the α_2_-peptides undergo transient intramolecular interactions to form a “closed” conformation, which makes the starting structures for the simulations presented here markedly more compact than the largely extended conformation of our previous 24-residue peptide models [40], [49].

#### Molecular dynamics simulations in water

**Figure 5:**
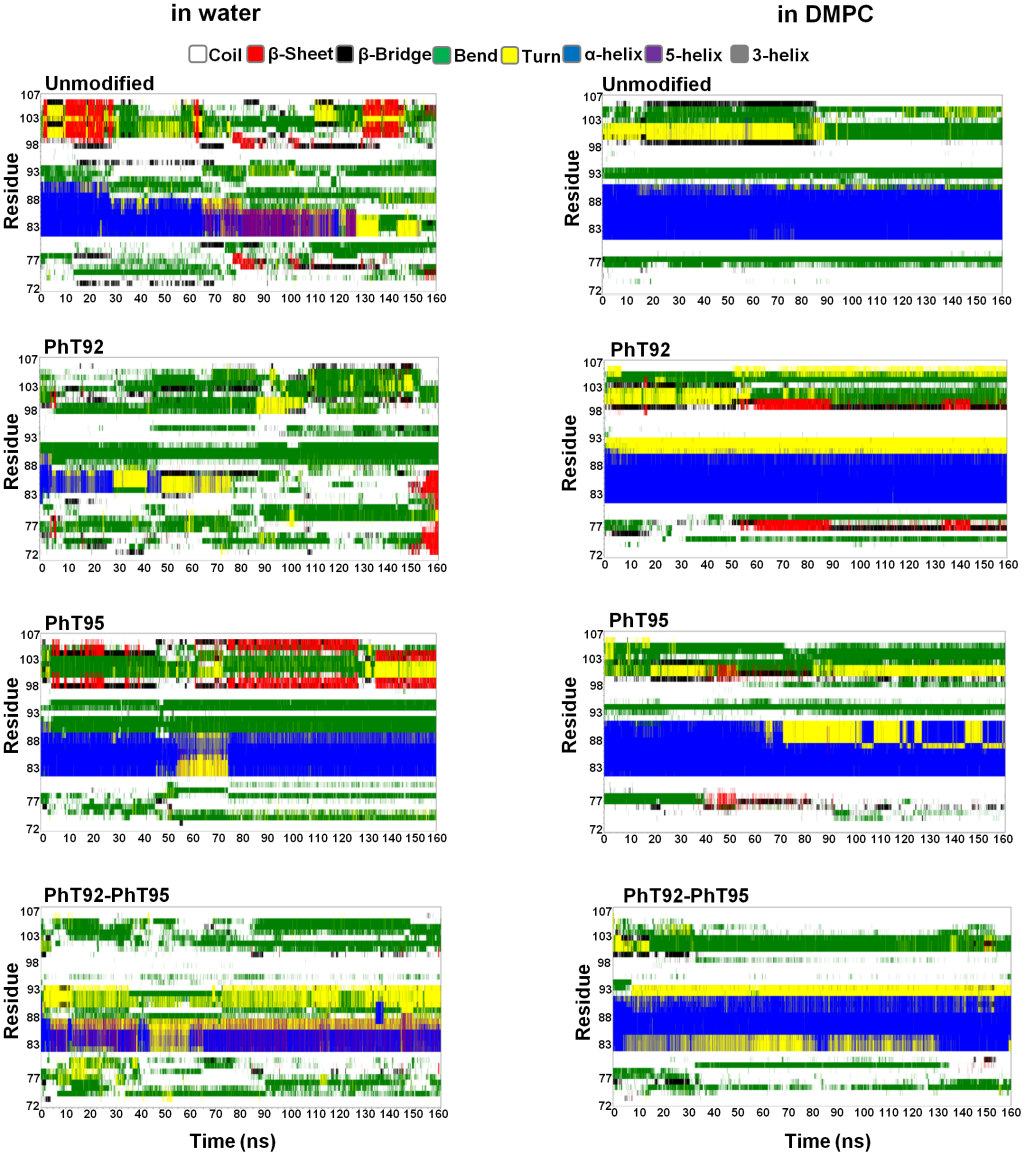
Secondary structure evolution of the MBP α_2_-peptides in water, and in a DMPC membrane environment. The α_2_-peptides (S72–S107) of myelin basic protein (MBP) were simulated for 160 ns using GROMACS 4.5.5 and the Gromos96 ffG53a6 force-field. The evolution in secondary structure at each frame was monitored using the dictionary of protein secondary structure (DSSP) algorithm [78]. Results for the unmodified peptide, the singly-phosphorylated α_2_-peptides (PhT92 and PhT95) and the doubly-phosphorylated α_2_-peptide (PhT92–PhT95), both in water and in a dimyristoylphosphatidylcholine (DMPC) membrane environment are shown as indicated.

**Figure 6:**
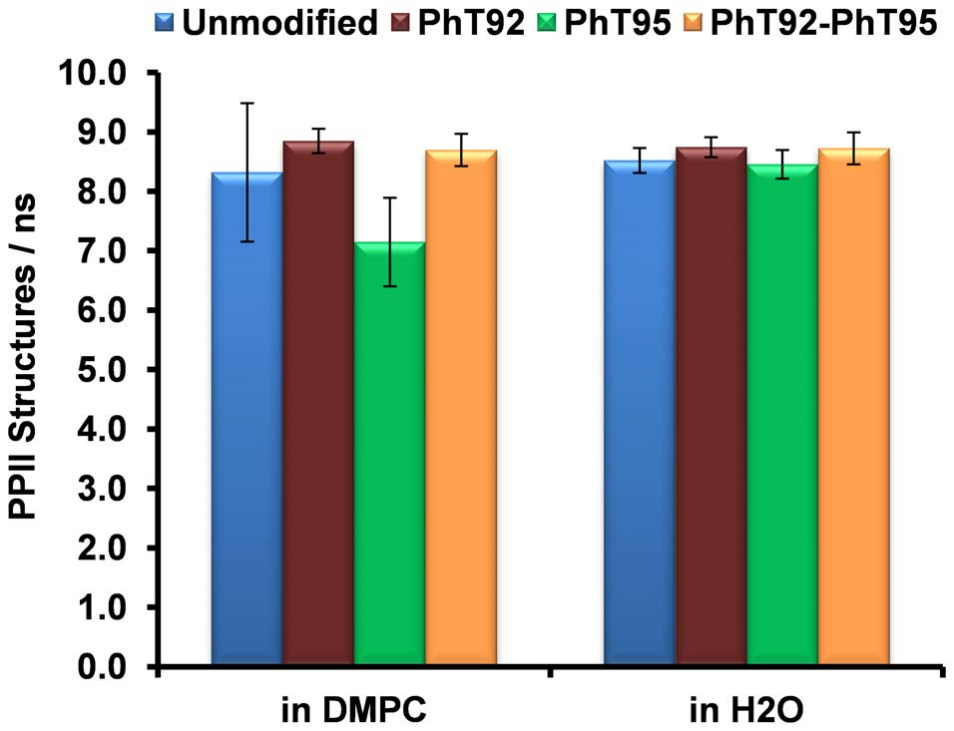
The number of PPII structures observed within the proline-rich region of the α_2_-peptide of MBP. The data were obtained through the analysis of molecular dynamics simulations of α_2_-peptides (S72–S107) of myelin basic protein (MBP) in water as well as in a dimyristoylphosphatidylcholine (DMPC) membrane bilayer system. This proline-rich region (T92-S99) is highly dynamic, and rapidly and reversibly transitions between a left-handed poly-proline II (PPII) structure and other conformations. The data that are presented represent the average frequency of observed PPII structures of at least 2 consecutive residues long within the proline-rich region. The error bars represent the average standard error from multiple independent simulations. In addition to the unmodified peptide, different phosphorylation variants were also analyzed: single phosphorylation at positions T92 and T95 (PhT92 and PhT95, respectively) and double phosphorylation (PhT92–PhT95).

In water, the MD experiments reveal that the α_2_-peptides are highly dynamic and the α-helix is unstable, as expected, based on Δ*G^H2O^* values (Table 3) and solution NMR data (Figure 2). As the simulations progress, significant portions of the α-helix becomes distorted, transitioning to π-helical, turn, or random coil conformations (Figure 5). All of the peptides, regardless of phosphorylation status, remain somewhat compact even as the α-helical segment unfolds, with P96, P97, and P98 generally being partially buried throughout the simulations. For the unmodified as well as phosphorylated peptides, α-helix unfolding tends to start at the C-terminus, and unfolding is most apparent in the PhT92- and PhT92-PhT95-α_2_-peptides (Figure 5). The residues N-terminal (S72-N81) and C-terminal (T92-S107) to the α-helical region (P82-I90) are highly dynamic. The N-terminal residues largely adopt coil and bend conformations throughout the simulations, whereas the C-terminal residues rapidly and reversibly transition between coil, bend, β-bridge, β-sheet, and PPII conformations (Figure 5, 6). The flexibility of the terminal regions also facilitates the formation of stabilizing electrostatic interactions between the negative phosphate group(s) and different basic residues within the phosphorylated peptides (see Discussion).

During the course of the MD experiments, the evolution of PPII structure in the proline-rich region, C-terminal to the α-helix, was analyzed quantitatively by observing the number of frames in which this structure is present. In this analysis, PPII structure is considered to be present if at least 2 consecutive residues adopted dihedral angles consistent with the PPII conformation threshold (as described in the Materials and Methods section). In the unmodified peptide, the average number of PPII conformations present is ∼8/ns with similar results for the phosphorylated variants (Figure 6). This observation indicates that phosphorylation may not have a significant effect on the formation of short PPII structures within the proline-rich region.

#### Molecular dynamics simulations on DMPC bilayers

**Figure 7:**
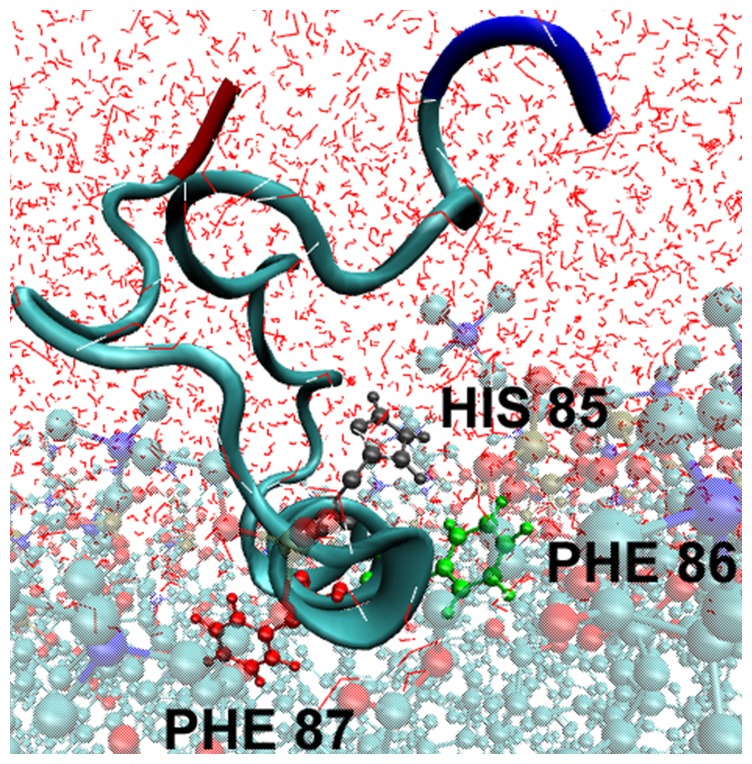
Positioning of the MBP α_2_-peptide relative to the DMPC membrane bilayer in molecular dynamics simulations. The N-terminal end of the α_2_-peptide (S72–S107) of myelin basic protein (MBP) is shown in red, and the C-terminal end is shown in blue. The α-helix was positioned relative to the dimyristoylphosphatidylcholine (DMPC) membrane bilayer (shown in cyan), so that the hydrophobic side-chains of F86 and F87 penetrated the bilayer, whereas hydrophilic residues such as H85 were positioned towards the solvent [20], [40], [51].

**Figure 8:**
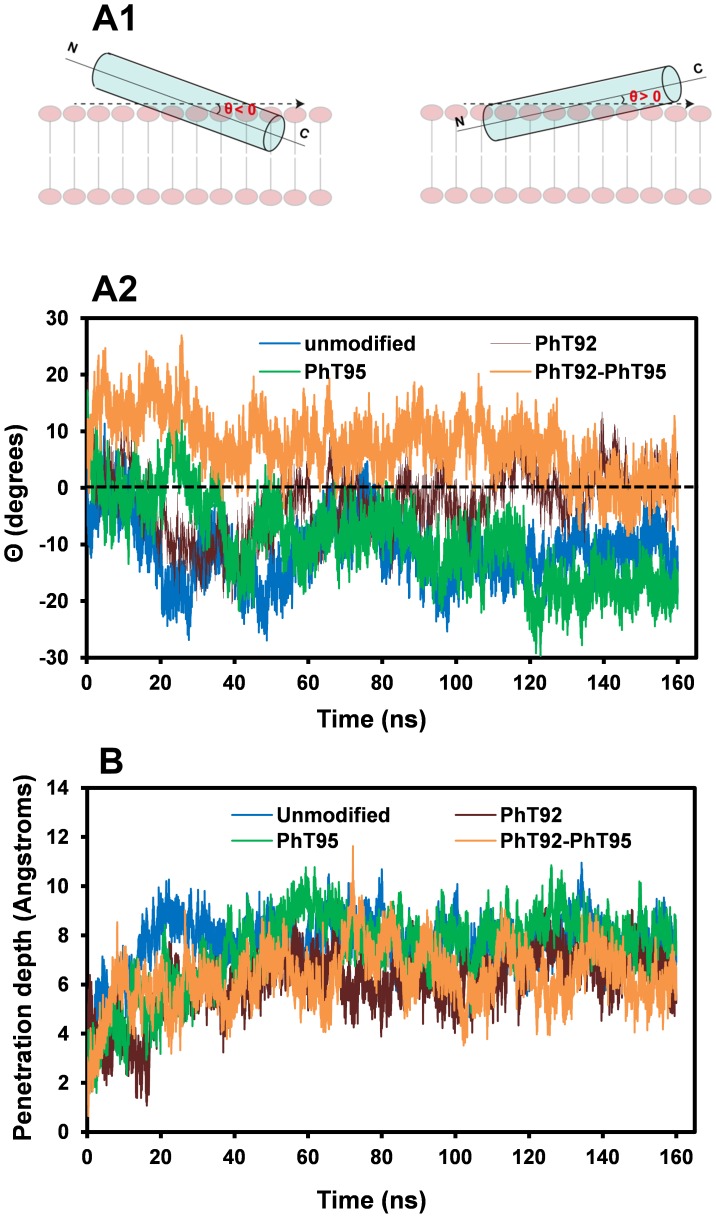
Change in tilt angle and membrane penetration depth of the α-helical segment in DMPC. Data were obtained from analysis of molecular dynamics experiments conducted in the presence of a dimyristoylphosphatidylcholine (DMPC) membrane bilayer for unmodified as well as singly- (PhT92, PhT95) and doubly- (PhT92–PhT95) phosphorylated α_2_-peptides of myelin basic protein (MBP). Panel A1 illustrates how the tilt angle Θ of the α-helix (residues P82-I90, represented as a cylinder) within the α_2_-peptide (S72–S107) was measured. Θ was determined as the angle between the axes passing through the geometric center of the helix and the axis that is parallel to the surface of the membrane. When Θ<0, the N-terminal portion of the helix is tilted away from the membrane surface (pointing upwards) whereas the C-terminal region is embedded (pointing downwards), and when Θ>0, the opposite is true. The evolution of the tilt angle was plotted as a function of simulation time over the entire experiment (A2). The change in penetration depth of the central α-helix into the DMPC bilayer for the unmodified α_2_-peptide, as well as the phosphorylated variants was also plotted (B). The penetration depth represents the distance between the center of mass of the α-helix and the surface of the membrane.

The four α_2_-peptides were also simulated for 160 ns in association with a DMPC bilayer. In these experiments, the starting peptide models used were the same as for the simulations in water. The peptides were initially placed on top of the bilayer, oriented with the hydrophobic residues F86 and F87 pointing towards the bilayer (Figure 7) [40], [49], [51]. At the beginning of the simulations, the phosphate groups in the phosphorylated peptides form electrostatic interactions with basic residues (See Discussion) and the central α-helical segment of all the peptides penetrates the bilayer with an average depth (Figure 8B) of ∼6–8 Å (measuring from the geometrical center of the α-helix to the phospholipid head groups located at the membrane surface). This membrane penetration greatly stabilizes the α-helix, allowing it to persist mostly intact throughout the entire simulation (Figure 5). In the unmodified peptide, as well as the single phosphorylated variants, any tendency for α-helical unfolding in DMPC appears to be confined to the last helical turn at the C-terminal end. In contrast, the α-helix of the PhT92–PhT95 doubly-phosphorylated peptide tends to undergo more significant unfolding at the N-terminal end of the helix, with a relatively slight perturbation at the C-terminal end (Figure 5).

A notable effect of phosphorylation is that it can alter the tilt of the helix in the DMPC membrane (Figure 8A). For the unmodified α_2_-peptide as well as the PhT95-variant, the α-helical segments (P82-I90) throughout the simulations are tilted such that the C-terminal portion of the helix is more submerged in the DMPC bilayer, whereas the N-terminal half is closer to the membrane surface (Θ<0, negative tilt). However, the helical tilt is different in the PhT92- and especially in the PhT92–PhT95-α_2_-peptide simulations, with the N-terminal half of the peptide tending to be more submerged into the bilayer, whereas the C-terminal half is at or above the membrane surface (Θ>0, positive tilt). The average helix penetration depth of ∼6 Å for the PhT92- and PhT92–PhT95-α_2_-peptides also tend to be slightly lower compared to the unmodified and PhT95-α_2_-peptides, which have penetration depths of ∼8 Å (Figure 8B).

The disordered regions that flank the α-helix remain mostly solvent-exposed, and are quite dynamic in all the α_2_-peptides in DMPC. There appears to be more transient formation of various structures (including β-bridge, β-sheet, bends, and PPII conformation) in the region C-terminal to the α-helix compared to the N-terminal disordered region (Figure 5). Similarly to the simulations in water, the observed number of PPII structures in the proline-rich region observed in the DMPC simulations is ∼8/ns for the unmodified as well as phosphorylated variants (Figure 6).

#### Molecular dynamics thermal-ramp simulations

In order to obtain information on thermal unfolding propensity of the α-helix within the α_2_-peptide in DMPC, thermal-ramp experiments were carried out in which the four α_2_-peptide variants were simulated for a total of 25 ns, with the experiment divided into three distinct steps: 10 ns with linear heating from 37°C to 500°C, followed by simulation for 5 ns at a constant temperature of 500°C, and finally by linear 10 ns cooling from 500°C back to 37°C. It should be noted that in these experiments, only the peptides were heated and the temperature of the bilayer was kept constant at 37°C, because the structure of the bilayer was observed to be severely and irreversibly disrupted at higher temperatures.

**Figure 9:**
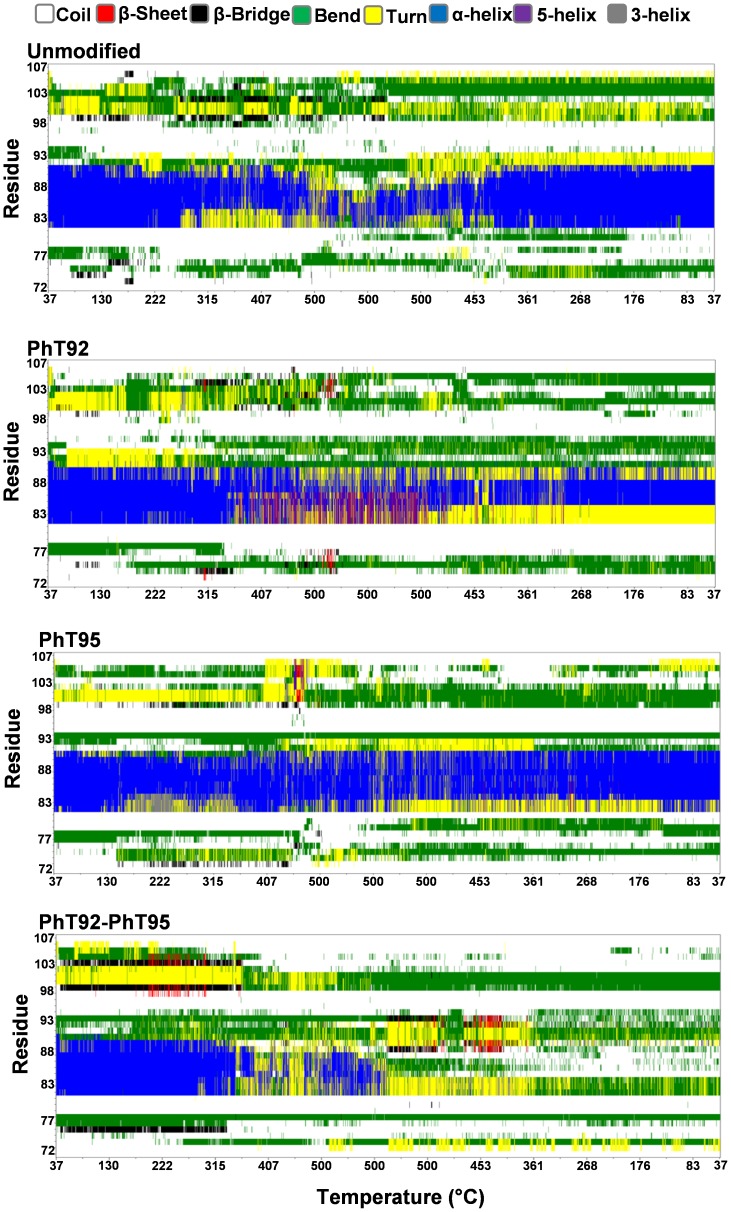
Evolution of secondary structure of MBP α_2_-peptides with temperature-ramping in a DMPC bilayer environment. The α_2_-peptides (S72–S107) of myelin basic protein (MBP) were simulated in a dimyristoylphosphatidylcholine (DMPC) membrane bilayer system using the simulated annealing protocol in GROMACS 4.5.5 and the Gromos96 ffG53a6 force-field. The peptide secondary structure was represented using the dictionary of protein secondary structure (DSSP) algorithm [78]. Data collected for the unmodified, singly-phosphorylated (PhT92, PhT95), and doubly-phosphorylated (PhT92–PhT95) α_2_-peptides were compared. In these *in silico* molecular dynamics experiments, the α_2_-peptides were heated linearly from 37°C to 500°C, over 10 ns. The temperature was held constant at 500°C for a further 5 ns before linearly cooling the peptide back to 37°C, over 10 ns.

In these temperature-ramp experiments, observed thermal unfolding of the α-helix of the unmodified α_2_-peptide starts at ∼250°C. Interestingly, unfolding is reversed when the peptide is cooled from 500°C to 37°C during the final 10 ns step (Figure 9). The PhT95-α_2_-peptide is apparently very stable to the effects of heat with only limited observed unfolding, primarily involving the first 2 residues at the N-terminal side of the helix. The α-helical unfolding in the PhT95-variant, however, is somewhat less reversible compared to the unmodified peptide. The α-helix of the PhT92-α_2_-peptide is observed to undergo more significant unfolding compared to either the unmodified α_2_-peptide or the PhT95-α_2_-peptide, with only the middle 4 residues of the α-helix retaining their starting conformation. Furthermore, observed α-helical unfolding in the PhT92-α_2_-peptide is almost totally irreversible. In thermal-ramp experiments, the α-helix of the PhT92–PhT95-α_2_-peptide is the only one that is observed to completely unfold, and this unfolding is totally irreversible. Overall, these data suggest that phosphorylation at position T92 in the α_2_-peptides reduces the apparent stability of the α-helix in a membrane environment.

## Discussion

### The Proline-rich Region of MBP is Disordered in Water

The proline-rich region in mammalian MBP (murine sequence –T92-P93-R94-T95-P96-P97-P98-S99-) is highly conserved (encoded by classic exon IV) and contains a minimal XP-X-XP SH3-ligand domain. We have previously demonstrated that MBP binds to several SH3-domains *in vitro*, and probably *in cellula*, including those of Fyn kinase, cortactin, and zonula occludens 1 (ZO-1) [24], [25], [48]. Co-expression of MBP with a constitutively-active form of Fyn-kinase caused extensive membrane elaboration, and branching complexity in cultured N19-oligodendrocyte membrane processes [24]. This phenotype was abolished by Pro-to-Gly substitutions at either proline residue within the XP-X-XP SH3-ligand consensus motif in MBP, thereby directly implicating this region in SH3-domain binding. Additionally, isothermal titration calorimetry experiments revealed that MBP variants with Pro-to-Gly substitutions within the proline-rich region, as well as pseudo-phosphorylation at the MAP-kinase sites (T92E and T95E), have reduced enthalpy upon binding to the SH3-domain of Fyn kinase [24]. More recently, using NMR spectroscopy, we have reported chemical shift perturbations within the proline-rich region when MBP α_2_-peptide was titrated with the SH3-domain of Fyn [49].

Given the strong experimental evidence suggesting that the proline-rich region is indeed an SH3-ligand, it is important to investigate its conformation and specifically to determine if this region spontaneously adopts PPII structure usually recognized by SH3-domains. Previously, we have shown by CD spectroscopy that an 18-residue peptide of MBP (F86-G103, murine sequence), containing the proline-rich region adopts some PPII structure in both aqueous buffer and with DPC micelles, that appears to be slightly stabilized from thermal denaturation by phosphorylation at T95 [20], [48]. However, given that there is an abundance of evidence that non-proline-rich regions within disordered conformations can adopt PPII structure [80], [90], we cannot un-ambiguously assign the observed PPII structure in the CD spectra to the proline-rich region due to potential contribution from the residues outside of this region. Thus, the NMR experiments reported here are important for conclusively determining the conformation of this proline-rich region *per se*.

The NMR experiments on the unmodified α_2_-peptide in water suggest that PPII structure is largely disfavored within the proline-rich region of MBP, with some PPII propensity (Figure 2B) only being present in the disordered regions at the N- and C-termini of the α_2_-peptide, according to the δ2D algorithm [68]. This observation is in contrast to the α_2_-peptide in DPC micelles [49], where the δ2D algorithm indicates that the proline-rich region has some propensity to form PPII structure (Figure 2C). Additionally, the secondary structure assignment methods XTLSSTR [91], PROSS [92], and SEGNO [93], all characterize the proline-rich region in the α_2_-peptide in DPC as adopting a PPII conformation (Figure 2D). These data thus suggest that PPII formation within the proline-rich region is highly sensitive to a lipid environment or, more likely, may be enhanced by the presence of the fully-formed adjacent α-helix.

The MD experiments allowed us to investigate the effects of phosphorylation on PPII formation in the proline-rich region. The MD results have to be interpreted with caution as the simulation time scale in these experiments is relatively short at 160 ns, due to limitations in computational resources presently available to us. Nevertheless, if we assume that the kinetics of unfolding/refolding of the PPII structure in the proline-rich region is similar for the unmodified and the phosphorylated variants, valuable insights can be gained in comparing the number of PPII structures observed during MD simulation experiments. The observation that phosphorylation does not significantly affect the formation of PPII structures of length 2-residues in the proline-rich region (Figure 6) suggests that any effect of phosphorylation on the binding of SH3-domains may only involve direct or indirect electrostatic effects (*vide infra*).

### The Central α-helix Stability is Reduced upon Phosphorylation

Disorder-to-order transitions are a common and often necessary feature of IDPs in order to interact with their binding partners, and these transitions play an important role in the multifunctionality of these proteins (*e.g.*, [22], [94]). The disordered regions or fragments within proteins that adopt defined secondary structure upon interaction with their binding partners have been termed molecular recognition fragments or elements (MoRFs or MoREs) [95]–[99]. The central (P82-I90) segment that is contained within the α_2_-peptides used in this study is an unequivocal α-helical molecular recognition fragment (α-MoRF). This region is disordered in water, but becomes α-helical upon interaction with phospholipids and other surfactants, or in the presence of membrane-mimetic solvents such as TFE ([23], [27], [51], [54], and references therein).

Similar to the methodology used for other IDPs and peptides [69], [86], [100]–[103], we have used equilibrium TFE-titration curves to evaluate the free energy change of the disorder-to-α-helical transition of the unmodified α_2_-peptide as well as three phosphorylated variants of it. The Δ*G^H2O^* of 14.9 kJ mole^−1^ for the disorder-to-α-helical transition of the unmodified α_2_-peptide indicates that, although α-helical formation is unfavourable in water, the free energy difference between the disordered and α-helical states is relatively modest, increasing the likelihood of a small population of the α-helical conformation in aqueous conditions.

**Figure 10:**
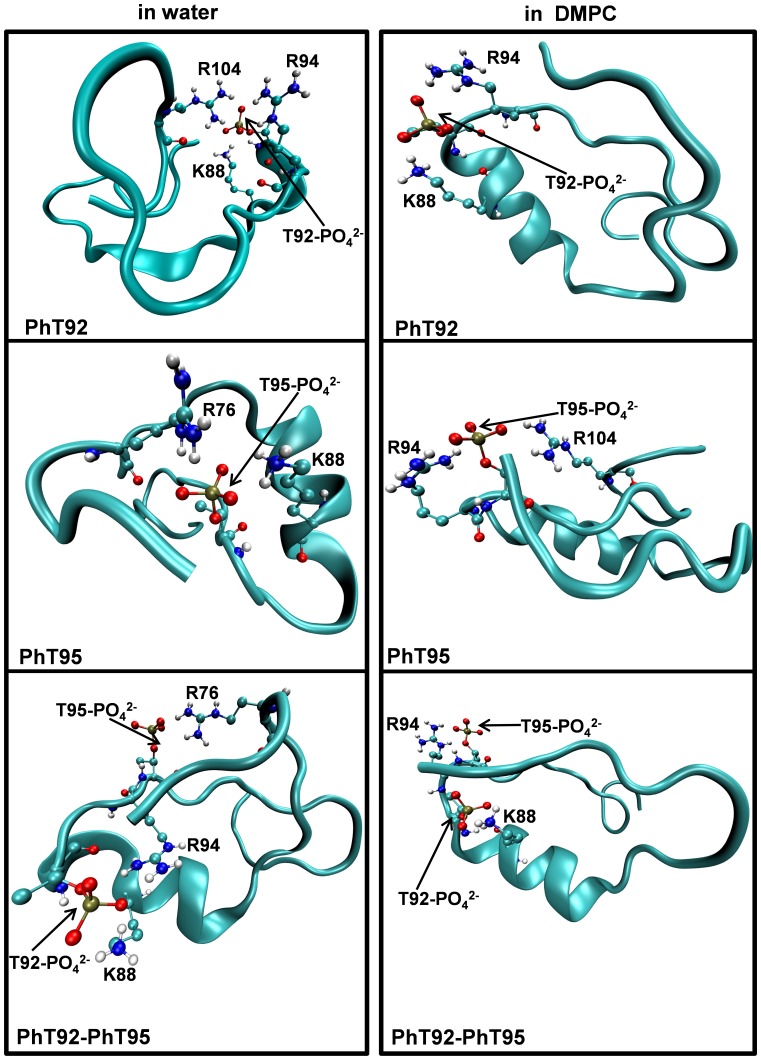
Structure of phosphorylated α_2_-peptides at the end of MD experiments in water and in DMPC. The dimyristoylphosphatidylcholine (DMPC) membrane bilayer and water molecules are not shown in order to improve clarity. The images are a representative snapshot of singly-phosphorylated (PhT92, PhT95), and doubly-phosphorylated (PhT92–PhT95) α_2_-peptides (S72–S107) of myelin basic protein (MBP) captured during the last 10 ns of the 160 ns molecular dynamics simulation experiments. Shown are examples of the type of electrostatic interactions between phosphorylated Thr residues and different basic residues within the α_2_-peptide variants.

The approach that we have adopted here allows for a systematic comparison of the effects of phosphorylation, at the two threonyl residues that lie just at or outside the C-terminal end of the α-helical region, on the stability of the α-helix. Phosphorylation has been shown to alter the stability of α-helices in other proteins in a position dependent manner [104]–[106]. Whereas phosphorylation close to the N-terminal end of an α-helix tends to be stabilizing, phosphorylation close to the C-terminal end of an α-helix can destabilize the helix due to electrostatic repulsion between the negative phosphate group and the α-helix dipole [104]. Consistent with this general principle, we find that the singly-phosphorylated MBP α_2_-peptide at position T92, as well as the doubly-phosphorylated α_2_-peptide at positions T92 and T95, both have markedly higher values of Δ*G^H2O^*, suggesting that the disorder-to-α-helical transition is less favoured. The singly-phosphorylated PhT95-α_2_-peptide also tends to have a higher Δ*G^H2O^* value compared to the unmodified α_2_-peptide, although this difference is smaller than for the two other phosphorylated α_2_-peptides. These results thus suggest that phosphorylation at the T92 position has a greater destabilizing effect on α-helical stability compared to phosphorylation at position T95. This conclusion is supported qualitatively by our thermal-ramp MD experiments (Figure 9), where the α-helix of the unmodified and PhT95-α_2_-peptides have higher apparent thermal stability, when associated with the DMPC membrane, than either of the PhT92- and PhT92–PhT95-α_2_-peptides. These results might be expected, given that T92 is closer to the α-helix (comprising residues P82-I90) than T95, being only two residues removed from the C-terminal end of the helix. Furthermore, our α_2_-peptide simulations in DMPC at physiological temperature demonstrate that an electrostatic interaction occurs between the phosphate group on T92 and the residue K88 in the middle of the central α-helix (see Figure 10 and below). Formation of this stable electrostatic interaction could cause strain in the α-helix, which could also contribute to destabilization. Given that attaining an α-helical conformation is essential for MBP-membrane interaction, the results suggest that phosphorylation could modulate 18.5-kDa MBP’s interaction with membranes by altering the energy landscape.

There have been other studies on intrinsically-disordered proteins or regions that suggest that C-terminal phosphorylation can inhibit the disorder-to-α-helical transition, and hence potentially compromise the effectiveness of α-MoRFs. For example, it has been reported that seryl phosphorylation at the C-terminal end of a region with α-helical propensity in the LD4 motif of paxillin inhibits the disorder-to-α-helical transition [107]. Similarly, it has been reported that seryl phosphorylation at the C-terminal end of an α-MoRF in the protein 4E-BP1 can modulate the free energy folding landscape, by shifting the disorder-to-order equilibrium towards the disordered species, regulating the interaction of 4E-BP1 with its partner el4E [108]. Hence, phosphorylation particularly at the C-terminal end of α-MoRFs may be an important and general molecular switching mechanism, regulating binding interactions by disfavouring the necessary disorder-to-order transition.

### Phosphorylation Alters the Orientation of the α-helix with Respect to the Membrane

Our previous electron paramagnetic resonance (EPR) and NMR experiments of full-length 18.5-kDa murine MBP had suggested that the α-helix within the molecular switch region of MBP is titled within the membrane such that the C-terminal end of the helix is submerged within the membrane, while the N-terminus of the helix is close to the membrane surface [51], [52], [54]. This tilt might be energetically favourable, in part, because it would enable the disordered, hydrophilic region N-terminal to the α-helix to be in the aqueous phase and not be buried within the lipid bilayer. Our previous MD studies on a 24-residue peptide of MBP (E80-G103, murine 18.5-kDa sequence), representing the molecular switch region and containing this α-helix within it, showed similar results to the EPR and NMR experiments. These simulations further suggested that phosphorylation reverses the tilt angle of the helix such that the N-terminus becomes more submerged in the membrane whereas the C-terminus is closer to the surface of the membrane [40].

The MD experiments presented here on the unmodified longer 36-residue α_2_-peptide (S72–S107), comfortably comprising the molecular switch region, also demonstrate that the α-helix is tilted within the DMPC membrane such that the C-terminal end of the helix is more submerged. However, the α-helix within the unmodified 36-residue α_2_-peptide has a less negative tilt angle (Θ) compared to the α-helix within the 24-residue peptide. The slight difference in the α-helical tilt between the 24- and 36-residue peptides could be due to the shorter α-helix in the 36-residue peptide (of length 9-residues versus 12-residues at the start of the simulation), and the presence of the longer disordered regions in the 36-residue peptide that flank the helix. In the 36-residue α_2_-peptide simulations, the central α-helix tilt angle Θ changes slightly upon single phosphorylation at T92, becoming less negative and approaching zero (Figure 8A). In the doubly-phosphorylated α_2_-peptide, the tilt angle Θ is actually positive, meaning that the N-terminus of the helix is more submerged in the bilayer compared to the C-terminus. An α-helical tilt angle near zero or positive allows the highly charged phosphate group at T92 to be further from the membrane surface and closer to water, enabling more efficient solvation, and easier formation of stabilizing electrostatic interactions with basic residues in the water-exposed disordered regions (see below). A more positive tilt angle Θ is less important in the case of PhT95 because the phosphate group is further away from the helix, and hence it is unsurprising that the tilt angle of the helix within this PhT95-α_2_-peptide is very similar to that of the unmodified α_2_-peptide. The differences in tilt angles mediated by phosphorylation could affect how the N- and C- terminal loop regions of this central α-helix are presented to the cytoplasm for binding to protein partners.

### Phosphorylation Modulates Electrostatic Interactions within the Molecular Switch Region of MBP

The molecular switch region of MBP, represented here by the 36-residue unmodified α_2_-peptide, has an overall net charge of +3 (assuming uncharged histidyl residues) with 2 acidic and 5 basic residues (Figure 1). Given that all but one of the basic residues is found outside the central α-helical segment, there is a greater potential for phosphate groups to be stabilized by electrostatic interactions, due to the flexibility of these largely disordered regions. One difference between unmodified and phosphorylated α_2_-peptides, in MD experiments conducted in water as well as in DMPC, is the pattern of electrostatic interactions observed. These electrostatic interactions are driven exclusively by the need to stabilize the −2 charge of each phosphate group. Whereas there are no observable stable electrostatic interactions that persist throughout the simulations in water for the unmodified α_2_-peptide, the phosphate groups in all three phosphorylated α_2_-peptides each form two or three electrostatic interactions. These electrostatic interactions reduce the conformational freedom of the peptides and tend to form early in the simulation, usually persisting throughout the simulations. In the PhT92-variant, these electrostatic interactions primarily involve the residues R94 and K88, and occasionally R104, with the side-chains of the residues clustering simultaneously around the phosphate group (Figure 10). In the PhT95-α_2_-peptide, electrostatic interactions occur between the phosphate group at T95, and two basic residues simultaneously (Figure 10). These interactions involve either K88 and R76, or R94 and R104. In the PhT92–PhT95 doubly-phosphorylated α_2_-peptide, a total of four electrostatic interactions occur, involving the two phosphate groups and basic residues in the peptide, with the phosphorylated T92 forming electrostatic interactions simultaneously with K88 and R94, whereas phosphorylated T95 forms electrostatic interactions with either R76 or R104. The R94 residue can also be re-oriented to form an electrostatic interaction with phosphorylated T95, leaving the phosphorylated T92 stabilized by just one electrostatic interaction.

In MD simulations conducted in DMPC, all the α_2_-peptides were observed to be far less dynamic compared to water. In the unmodified α_2_-peptide, an electrostatic interaction occurs between E80, in the N-terminal disordered region, and K88 in the middle of the α-helix that appears to be of only moderate stability, tending to be only transiently formed throughout the simulation. This electrostatic interaction partially restrains the N-terminal disordered region, and may play a role in ensuring a correct orientation for binding interactions. This electrostatic interaction is absent in the phosphorylated α_2_-peptides, due in part to the preference of the K88 side-chain to interact with the phosphate groups. During the simulation of the PhT92 peptide in DMPC, we observed electrostatic interactions between the phosphate group at T92 and K88, as well as with R94 (Figure 10). The third electrostatic interaction between the phosphate group at T92 and R104 that occurs in water is not present in the DMPC condition, due to the C-terminal region being less dynamic. In contrast to the PhT92 α_2_-peptide, the electrostatic interactions of PhT95 in DMPC do not involve K88. Rather, the phosphate group in PhT95 is stabilized mainly by interaction with the guanidinium group of the neighboring R94 residue, and sometimes simultaneously with the R104 residue (Figure 10). The absence of an electrostatic interaction involving K88 in the PhT95-α_2_-peptide may help to account for the increased α-helical stability in this peptide due to reduced strain (see above). In the simulation experiments of PhT92–PhT95 in DMPC, distinctly different patterns of electrostatic interactions were observed. One of the observed patterns involve the phosphate groups at T92 and T95 being stabilized by electrostatic interactions with K88 and R94, respectively (Figure 10). A second pattern involves phosphorylated T92 and T95 interacting with R94 and R104, respectively. These distinct patterns of electrostatic interactions may represent separate ensemble structures stabilized by double-phosphorylation in the molecular switch region of MBP.

Overall, the differences that we see in electrostatic interactions as a result of differing phosphorylation status may be a mechanism for controlling the conformation of the disordered regions in the molecular switch of MBP. This may be important in properly orientating these disordered regions in the cytoplasm for more specific protein-protein interactions.

## Conclusions

In this study, we have investigated the structure, stability, and dynamics of a key, highly-conserved central segment within the 18.5-kDa isoform of MBP, containing a proline-rich region with two MAP-kinase phosphorylation sites adjacent to a region that can form an amphipathic, membrane-anchoring α-helix. The finding that the proline-rich region adopts PPII structure in the presence of DPC lysophospholipids [49] when the adjacent α-helix is formed, but not under aqueous conditions when the helical region is disordered, suggests that the α-helical and PPII regions may be cooperatively linked. This phenomenon could be a mechanism for ensuring that SH3-domains, which typically recognize PPII structure, bind to MBP only when the protein is anchored to the membrane via its central α-helix, and not free in the cytoplasm (see also [29], [40]).

Phosphorylation at the MAP-kinase sites has marked effects on the stability and conformation of the conserved central segment of MBP. Phosphorylation alters the energy landscape of the central segment by disfavoring α-helical formation, which could inhibit MBP-membrane association. Phosphorylation also appears to play a direct role in MBP-membrane interaction by altering both the tilt angle and the penetration depth of the helix in the membrane. Phosphorylation can also restrain the highly-dynamic regions that flank the α-helix through formation of local electrostatic interactions. This post-translational modification may be important in orientating these highly-dynamic regions for binding reactions. Additionally, the restriction in conformational freedom resulting from the formation of these electrostatic interactions may play a role in facilitating binding-induced folding mechanisms that are often critical in IDPs, by reducing the conformational entropy of the unbound state. Overall, the results presented here support the hypothesis that the central conserved region in MBP represents an important molecular switch during myelinogenesis and turnover, and illustrates possible mechanisms of control through phosphorylation/dephosphorylation at the MAP-kinase sites in MBP.

## Supporting Information

Figure S1 **C_α_ chemical shift index plot of residues in the unmodified α_2_-peptide (S72–S107) of myelin basic protein.** Random coil values have been adjusted for sequence dependence. The horizontal reference lines correspond to the threshold values, above which any deviations begin to reflect tendency to form α- or β- structures (positive and negative differences, respectively).
(TIF)

Figure S2 **Secondary structure propensity (SSP) score calculated for the unmodified α_2_-peptide (S72–S107) of myelin basic protein.** The scores are based on the C_α_, C_β_, and H_α_ chemical shifts assigned in the presence of dodecylphosphocholine (DPC) micelles (red, PDB ID 2LUG), and in aqueous solution (black). The results show the lack of α- or β-structure propensity when the peptide is in aqueous solution. The algorithm was created and distributed by the Forman-Kay group [84].
(TIF)

Figure S3 **CD spectra of α_2_-peptide variants (S72–S107) of myelin basic protein (MBP) at different concentrations of trifluoroethanol (TFE).** Data are shown for unmodified as well as singly- (PhT92, PhT95) and doubly- (PhT92–PhT95) phosphorylated myelin basic protein α_2_-peptides. Solutions contained 100 µM peptide, 20 mM HEPES-NaOH, pH 7.4 with trifluoroethanol (TFE) concentrations of 0, 1.4, 2.8, 4.2 and 5.6 M as indicated. The samples were incubated at 25°C for 16 hours before scanning. The CD scans were collected using a Jasco J-815 spectropolarimeter (Japan Spectroscopic, Tokyo, Japan) using a quartz demountable cuvette with a 0.5-mm path length with thermostatting at 25°C using a Jasco PTC-424S/15 Peltier temperature controller (Japan Spectroscopic, Tokyo, Japan). Each scan was collected at a scan rate of 100 nm/min, and represents an average of 10 scans. Corresponding buffer scans were also collected and were subtracted from sample scans before presentation of the data. The data presented are normalized to mean residue ellipticity.
(TIF)

Figure S4 **Validation of temperature ramp (simulated annealing) molecular dynamics simulation experiments.** The 9-helical residues (P82-I90) of the myelin basic protein (MBP) 36-residue α_2_-peptide were all mutated to (A) alanine, (B) valine and (C) glycine respectively. The three mutated peptides were subsequently subjected to the simulated annealing protocol (see Materials and Methods) in a dimyristoylphosphatidylcholine (DMPC) membrane bilayer system using GROMACS 4.5.5 and the Gromos96 ffG53a6 force-field. The evolution of peptide secondary structure was represented using the dictionary of protein secondary structure (DSSP) algorithm [78]. In these simulated annealing experiments, the peptides were heated linearly from 37°C to 500°C, over 10 ns. The temperature was held constant at 500°C for a further 5 ns before linearly cooling the peptide back to 37°C, over 10 ns. The results indicate that the poly-alanine α-helix has the highest apparent thermal stability with the helix staying almost fully intact up to ∼1 ns after the temperature reaches 500°C. The poly-valine α-helix becomes significantly shorter at a temperature less than 500°C and is completely abolished upon reaching 500°C, while the poly-glycine α-helix is observed to completely unfold well before 500°C. The results are consistent with the measured helical propensity of the respective amino acids which have a rank order of alanine>valine>glycine [77].
(TIF)

Table S1 **The ^1^H, ^15^N and ^13^C chemical shifts of the α_2_-peptide of MBP.** Data were aquired in aqueous solution and were referenced to DSS (2,2-dimethylsilapentane-5-sulphonic acid). Chemical shifts were deposited to the Biological Magnetic Resonance Bank (ID number 19186).
(PDF)

Table S2 **The TALOS+ dihedral angle prediction results, with a classification for each prediction.**
(PDF)
