# Correction: The Effects of Threonine Phosphorylation on the Stability and Dynamics of the Central Molecular Switch Region of 18.5-kDa Myelin Basic Protein

**DOI:** 10.1371/journal.pone.0131653

**Published:** 2015-07-01

**Authors:** The PLOS ONE Staff

Fig 2 is missing from the PDF version of this paper. The publisher apologizes for the error. Please see Fig 2 in the online version of the article or below.

**Fig 2:**
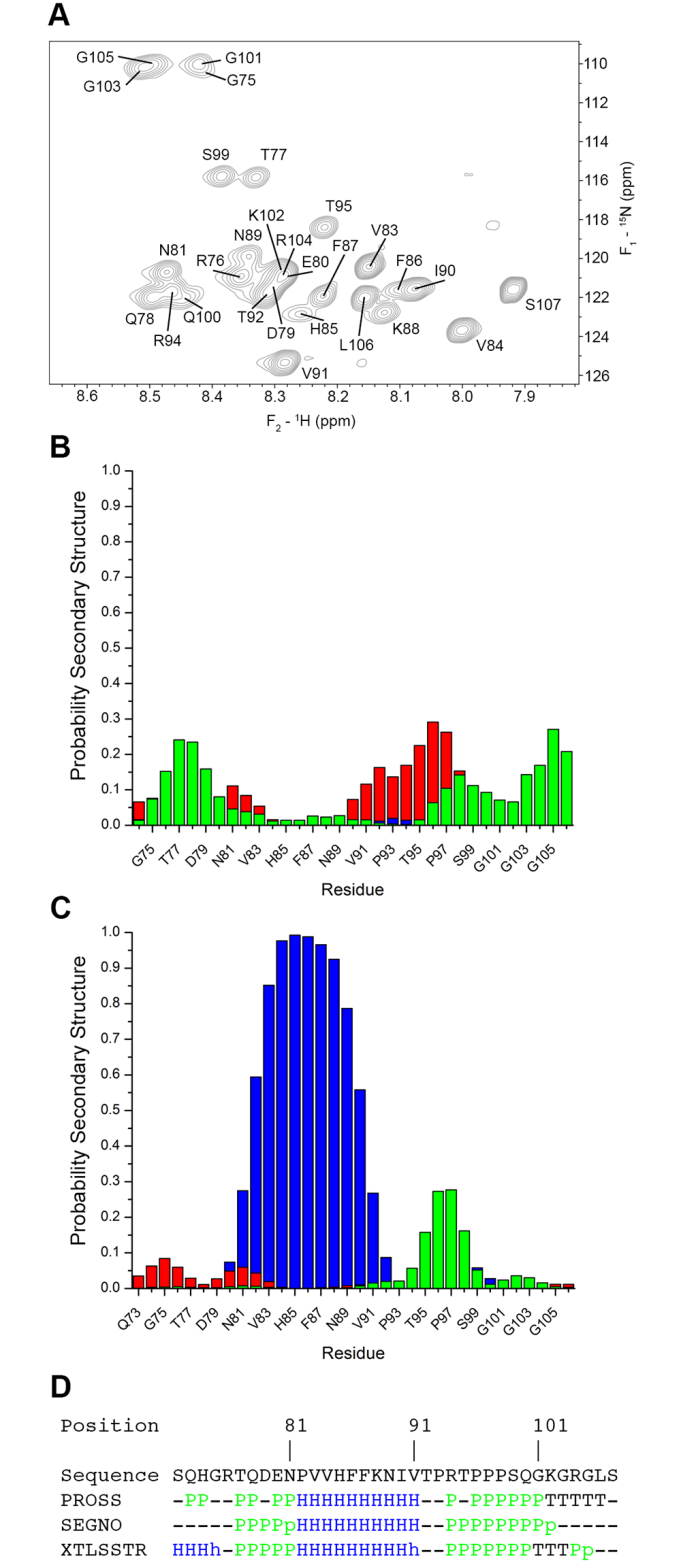
Nitrogen-HSQC NMR spectra and secondary structure analysis of the MBP α_2_-peptide. (A) The ^1^H-^15^N HSQC spectrum of uniformly ^13^C-^15^N-labelled α_2_-peptide (S72-S107) dissolved in 20 mM HEPES-NaOH, 100 mM NaCl, and 10% D_2_O at a concentration of 1.47 mM, and recorded at 295 K. A total of 29 of 31 expected backbone peaks were assigned (there are 5 prolyl residues). The HSQC spectrum was processed by applying a 90°-shifted squared-sine bell function, and zero-filled up to 256 and 2048 complex points along *F*_1_ and *F*_2_, respectively, prior to Fourier transformation using NMRPipe. (B) Prediction of secondary structure probabilities for each residue (populations per residue) in the α_2_-peptide in aqueous solution, using a method designed for disordered proteins [68]. The method differentiates between α-helix (blue), β-sheet (red), PPII (green), and random coil (not shown). The probabilities were calculated using the H_α_, H_N_, C_α_, C_β_, C’, and N chemical shift assignments for the α_2_-peptide. (C) Prediction of secondary structure probabilities for each residue in the α_2_-peptide in the presence of DPC, using a method designed for disordered proteins [49,68]. The H_α_, H_N_, C_α_, C_β_, C’, and N chemical shift assignments deposited in BMRB 6100 were used to calculate the secondary structure probabilities. (D) Secondary structure assignment methods used on the α_2_-peptide in the presence of DPC (PDB ID 2LUG). The XTLSSTR [91], PROSS [92], and SEGNO [93] methods all take into consideration PPII conformations, and differentiate them from coil, α, and β structures. The PROSS and SEGNO results were calculated using the “*Polyproline*” server created by the DSIMB bioinformatics team. The XTLSSTR results were obtained via the 2struc server created by the Wallace Laboratory [109]. The assignment indicates that the proline-rich region has a PPII conformation.
