# Supplementary figures and images for: The Effects of Threonine Phosphorylation on the Stability and Dynamics of the Central Molecular Switch Region of 18.5-kDa Myelin Basic Protein
Source: PLoS One. 2013 Jul 5;8(7):e68175. doi: 10.1371/journal.pone.0068175 (PMC3702573; doi:10.1371/journal.pone.0068175)

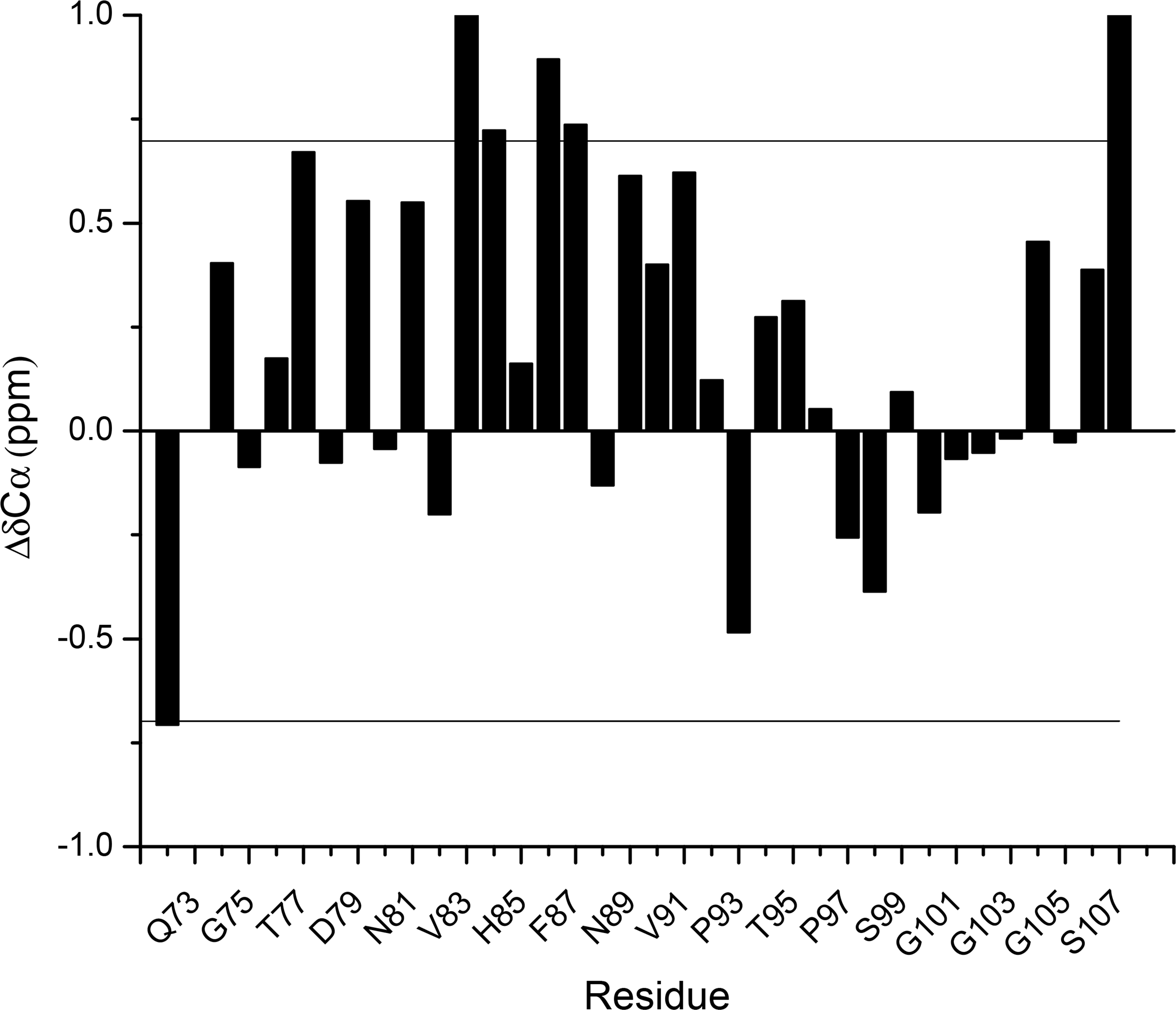

Supplement: Figure S1 — Cα chemical shift index plot of residues in the unmodified α2-peptide (S72–S107) of myelin basic protein. Random coil values have been adjusted for sequence dependence. The horizontal reference lines correspond to the threshold values, above which any deviations begin to reflect tendency to form α- or β- structures (positive and negative differences, respectively). (TIF) [file pone.0068175.s001.tif]

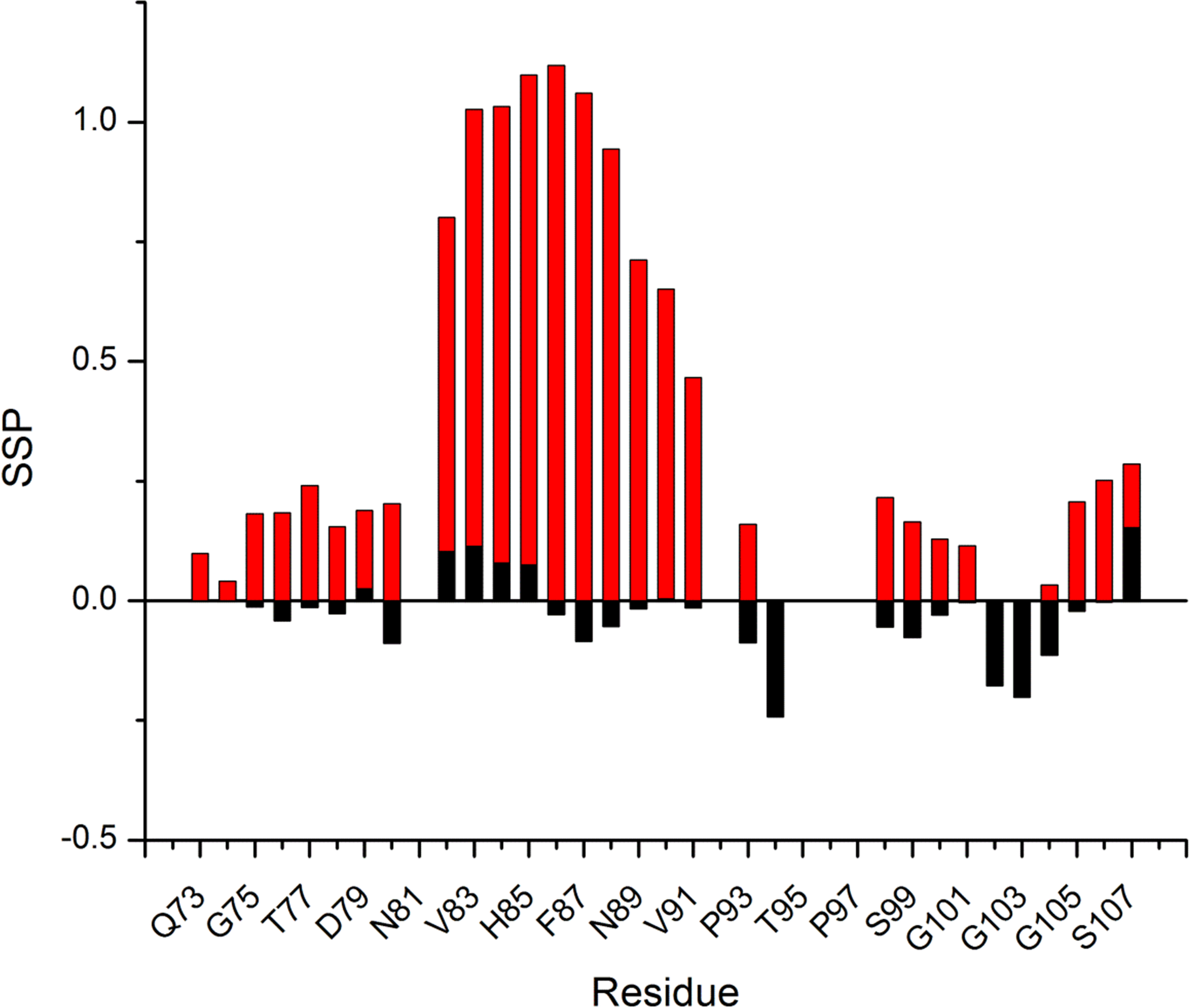

Supplement: Figure S2 — Secondary structure propensity (SSP) score calculated for the unmodified α2-peptide (S72–S107) of myelin basic protein. The scores are based on the Cα, Cβ, and Hα chemical shifts assigned in the presence of dodecylphosphocholine (DPC) micelles (red, PDB ID 2LUG), and in aqueous solution (black). The results show the lack of α- or β-structure propensity when the peptide is in aqueous solution. The algorithm was created and distributed by the Forman-Kay group [84]. (TIF) [file pone.0068175.s002.tif]

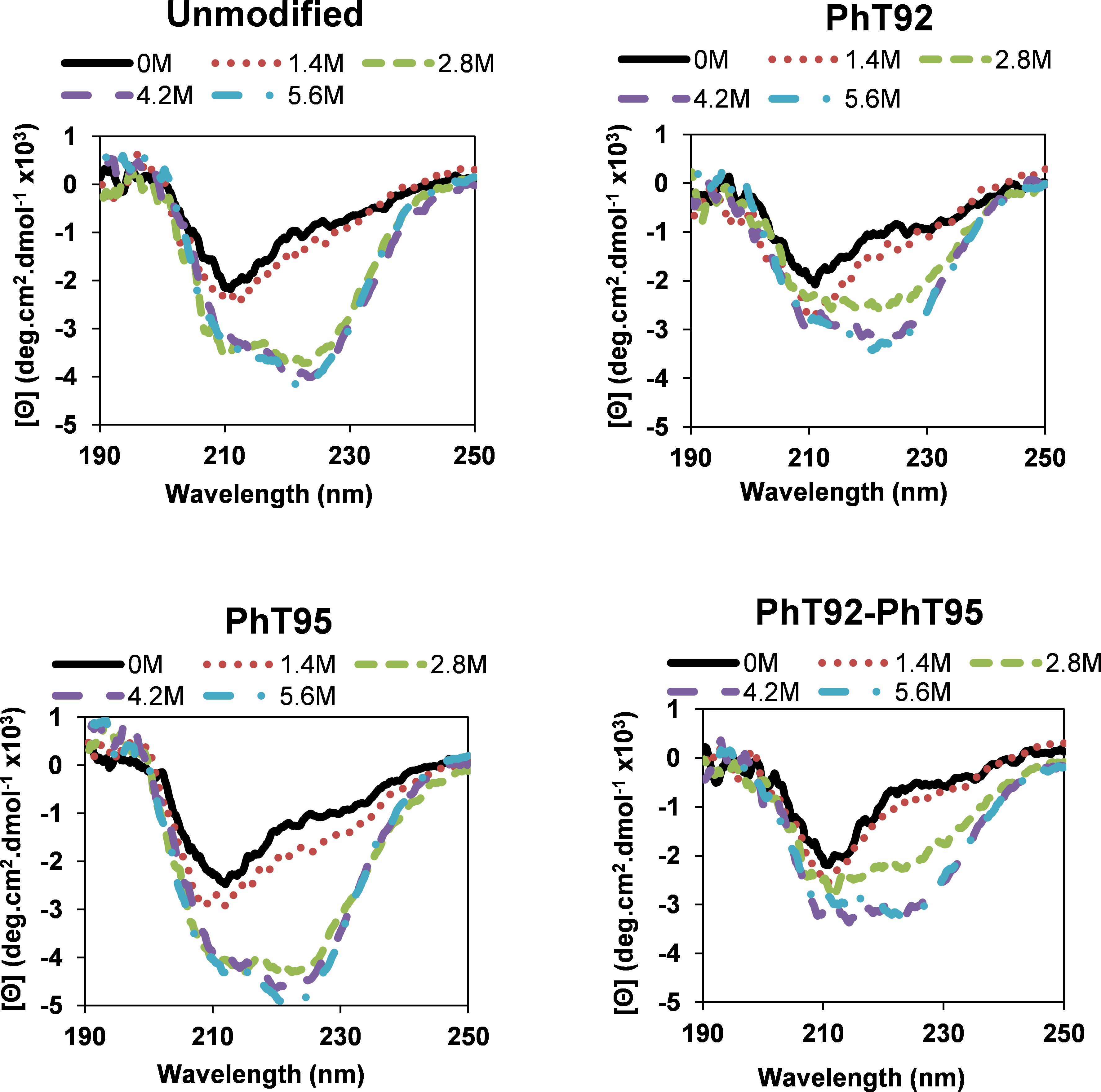

Supplement: Figure S3 — CD spectra of α2-peptide variants (S72–S107) of myelin basic protein (MBP) at different concentrations of trifluoroethanol (TFE). Data are shown for unmodified as well as singly- (PhT92, PhT95) and doubly- (PhT92–PhT95) phosphorylated myelin basic protein α2-peptides. Solutions contained 100 µM peptide, 20 mM HEPES-NaOH, pH 7.4 with trifluoroethanol (TFE) concentrations of 0, 1.4, 2.8, 4.2 and 5.6 M as indicated. The samples were incubated at 25°C for 16 hours before scanning. The CD scans were collected using a Jasco J-815 spectropolarimeter (Japan Spectroscopic, Tokyo, Japan) using a quartz demountable cuvette with a 0.5-mm path length with thermostatting at 25°C using a Jasco PTC-424S/15 Peltier temperature controller (Japan Spectroscopic, Tokyo, Japan). Each scan was collected at a scan rate of 100 nm/min, and represents an average of 10 scans. Corresponding buffer scans were also collected and were subtracted from sample scans before presentation of the data. The data presented are normalized to mean residue ellipticity. (TIF) [file pone.0068175.s003.tif]

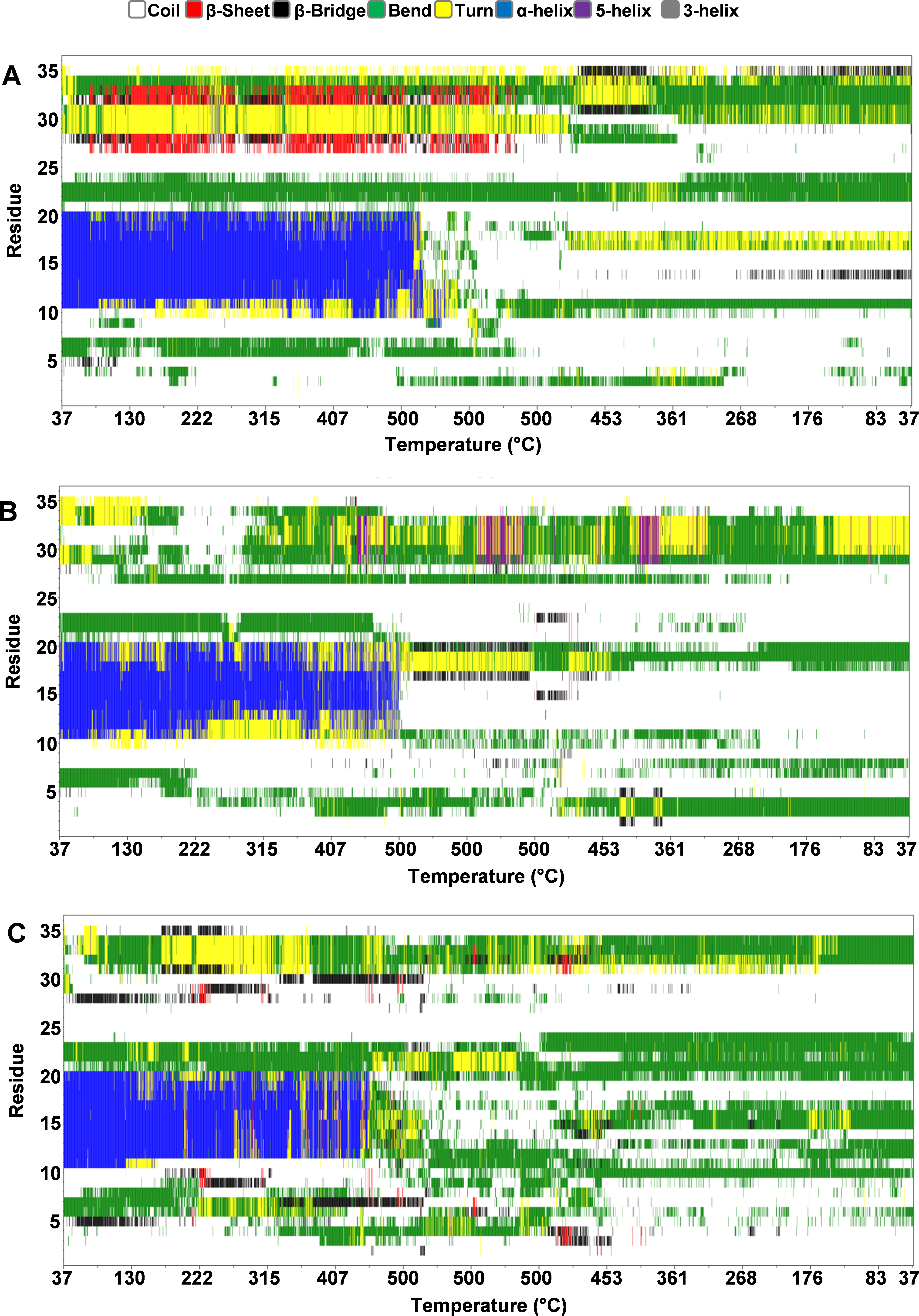

Supplement: Figure S4 — Validation of temperature ramp (simulated annealing) molecular dynamics simulation experiments. The 9-helical residues (P82-I90) of the myelin basic protein (MBP) 36-residue α2-peptide were all mutated to (A) alanine, (B) valine and (C) glycine respectively. The three mutated peptides were subsequently subjected to the simulated annealing protocol (see Materials and Methods) in a dimyristoylphosphatidylcholine (DMPC) membrane bilayer system using GROMACS 4.5.5 and the Gromos96 ffG53a6 force-field. The evolution of peptide secondary structure was represented using the dictionary of protein secondary structure (DSSP) algorithm [78]. In these simulated annealing experiments, the peptides were heated linearly from 37°C to 500°C, over 10 ns. The temperature was held constant at 500°C for a further 5 ns before linearly cooling the peptide back to 37°C, over 10 ns. The results indicate that the poly-alanine α-helix has the highest apparent thermal stability with the helix staying almost fully intact up to ∼1 ns after the temperature reaches 500°C. The poly-valine α-helix becomes significantly shorter at a temperature less than 500°C and is completely abolished upon reaching 500°C, while the poly-glycine α-helix is observed to completely unfold well before 500°C. The results are consistent with the measured helical propensity of the respective amino acids which have a rank order of alanine>valine>glycine [77]. (TIF) [file pone.0068175.s004.tif]
