# Supplementary material for: The Effects of Threonine Phosphorylation on the Stability and Dynamics of the Central Molecular Switch Region of 18.5-kDa Myelin Basic Protein
Source: PLoS One. 2013 Jul 5;8(7):e68175. doi: 10.1371/journal.pone.0068175 (PMC3702573; doi:10.1371/journal.pone.0068175)
Supplement: Table S1 — The 1H, 15N and 13C chemical shifts of the α2-peptide of MBP. Data were aquired in aqueous solution and were referenced to DSS (2,2-dimethylsilapentane-5-sulphonic acid). Chemical shifts were deposited to the Biological Magnetic Resonance Bank (ID number 19186). (PDF) [file pone.0068175.s005.pdf]

Table S1. The ^{1}H, ^{15}N and ^{13}C chemical shifts of the α_2-peptide of MBP

Residue	H_α	C_α	C_β	C'	N	H_N
S72	4.002	57.533	63.876	--	121.247	--
Q73	--	--	--	--	--	--
H74	4.633	56.224	30.306	--	120.347	--
G75	3.955	45.203	--	173.081	110.436	8.403
R76	4.451	56.085	30.875	175.894	120.885	8.348
T77	4.319	62.161	69.778	173.769	115.807	8.321
Q78	4.357	55.994	29.243	174.644	121.96	8.492
D79	4.553	54.543	41.122	175.019	121.471	8.3
E80	4.319	56.357	30.395	175.013	120.885	8.28
N81	4.938	51.46	38.855	172.081	120.69	8.465
P82	4.414	63.289	32.073	--	136.347	--
V83	3.969	62.914	32.199	175.144	120.397	8.143
V84	3.978	62.383	32.547	174.644	123.62	7.999
H85	4.543	55.632	30.422	173.456	122.839	8.252
F86	4.526	57.624	39.669	174.159	121.53	8.1
F87	4.538	57.577	39.638	174.201	121.862	8.218
K88	4.2	56.239	33.052	174.644	122.741	8.122
N89	4.627	53.274	38.765	173.769	119.909	8.334
I90	4.164	61.07	38.675	175.081	121.471	8.074
V91	4.14	62.161	32.598	175.033	125.28	8.28
T92	4.565	59.712	69.687	171.69	121.764	8.307
P93	4.402	63.095	32.073	--	138.479	--
R94	4.338	55.994	30.694	175.394	121.569	8.458
T95	4.552	59.712	69.687	171.269	118.346	8.218
P96	4.66	61.502	30.643	--	140.458	--
P97	4.696	61.293	30.643	--	136.809	--
P98	4.43	63.103	31.989	--	135.378	--
S99	4.385	58.443	63.793	173.753	115.71	8.382
Q100	4.363	55.904	29.424	175.394	122.057	8.444
G101	3.947	45.203	--	173.144	109.948	8.417
K102	4.326	56.357	30.422	176.107	120.69	8.287
G103	3.954	45.232	--	173.081	110.143	8.506
R104	4.335	56.425	32.892	176.17	120.831	8.285
G105	3.955	45.203	--	173.081	110.017	8.492
L106	4.411	55.178	42.392	175.769	121.96	8.15
S107	4.247	59.984	64.881	177.581	121.569	7.917

Data were aquired in aqueous solution and were referenced to DSS (2,2-dimethylsilapentane-5-sulphonic acid). Chemical shifts were deposited to the Biological Magnetic Resonance Bank (ID number 19186).
