# Supplementary material for: The Effects of Threonine Phosphorylation on the Stability and Dynamics of the Central Molecular Switch Region of 18.5-kDa Myelin Basic Protein
Source: PLoS One. 2013 Jul 5;8(7):e68175. doi: 10.1371/journal.pone.0068175 (PMC3702573; doi:10.1371/journal.pone.0068175)
Supplement: Table S2 — The TALOS+ dihedral angle prediction results, with a classification for each prediction. (PDF) [file pone.0068175.s006.pdf]

Table S2. TALOS+ dihedral angle prediction results, with a classification for each prediction

Residue	ϕ	ψ	Classification
S72	--	--	None
Q73	-138	139	Dyn
H74	-61	137	Dyn
G75	90	-3	Dyn
R76	-55	135	Dyn
T77	-67	136	Dyn
Q78	-106	-9	Dyn
D79	-95	139	Warn
E80	-115.968	139.802	Dyn
N81	-96.848	127.87	Dyn
P82	-68.453	149.187	Dyn
V83	-110.601	128.118	Dyn
V84	-90.481	127.522	Dyn
H85	-89.713	127.267	Dyn
F86	-87.745	138.129	Dyn
F87	-98.921	134.646	Dyn
K88	-86.982	141.798	Dyn
N89	-79.489	128.297	Dyn
I90	-95.929	125.066	Dyn
V91	-98.624	131.607	Dyn
T92	-107.254	137.364	Good
P93	-66.606	147.445	Dyn
R94	-97.716	135.575	Good
T95	-103.733	126.211	Good
P96	-71.619	145.452	Good
P97	-68.431	152.049	Good
P98	-65.779	151.533	Dyn
S99	-95.467	130.095	Dyn
Q100	-105.465	-5.803	Dyn
G101	-5.105	-177.708	Dyn
K102	-99.839	-6.351	Dyn
G103	92.895	-4.619	Dyn
R104	-78.399	147.868	Dyn
G105	88.01	175.653	Dyn
L106	-93.522	121.054	Dyn
S107	--	--	None
